# Borate-Based Compounds as Mixed Polyanion Cathode Materials for Advanced Batteries

**DOI:** 10.3390/molecules27228047

**Published:** 2022-11-19

**Authors:** Giancarlo Dominador D. Sanglay, Jayson S. Garcia, Mecaelah S. Palaganas, Maurice Sorolla, Sean See, Lawrence A. Limjuco, Joey D. Ocon

**Affiliations:** 1 Laboratory of Electrochemical Engineering (LEE), Department of Chemical Engineering, University of the Philippines Diliman, Quezon City 1101, Philippines; 2 Energy Engineering Program, National Graduate School of Engineering, College of Engineering, University of the Philippines Diliman, Quezon City 1101, Philippines; 3 DOST-NICER Advanced Batteries Center, University of the Philippines Diliman, Quezon City 1101, Philippines; 4 Department of Chemical Engineering, University of the Philippines Diliman, Quezon City 1101, Philippines; 5 Institute of Chemistry, University of the Philippines Diliman, Quezon City 1101, Philippines; 6 College of Engineering, University of Southeastern Philippines, Obrero, Davao City 8000, Philippines

**Keywords:** cathode, battery, mixed polyanion, borophosphate, borosulfate, borosilicate

## Abstract

Rational design of new and cost-effective advanced batteries for the intended scale of application is concurrent with cathode materials development. Foundational knowledge of cathode materials’ processing–structure–properties–performance relationship is integral. In this review, we provide an overview of borate-based compounds as possible mixed polyanion cathode materials in organic electrolyte metal-ion batteries. A recapitulation of lithium-ion battery (LIB) cathode materials development provides that rationale. The combined method of data mining and high-throughput ab initio computing was briefly discussed to derive how carbonate-based compounds in sidorenkite structure were suggested. Borate-based compounds, albeit just close to stability (viz., <30 meV at^−1^), offer tunability and versatility and hence, potential effectivity as polyanion cathodes due to (1) diverse structures which can host alkali metal intercalation; (2) the low weight of borate relative to mature polyanion families which can translate to higher theoretical capacity; and a (3) rich chemistry which can alter the inductive effect on earth-abundant transition metals (e.g., Ni and Fe), potentially improving the open-circuit voltage (OCV) of the cell. This review paper provides a reference on the structures, properties, and synthesis routes of known borate-based compounds [viz., borophosphate (BPO), borosilicate (BSiO), and borosulfate (BSO)], as these borate-based compounds are untapped despite their potential for mixed polyanion cathode materials for advanced batteries.

## 1. Introduction

Energy storage technology has accelerated the progress in our pursuit of sustainable development, particularly in portable applications, such as electric vehicles, and in large-scale stationary uses, such as renewable energy systems. As volumetric demands and performance requirements for energy storage increase [1], technological improvements are integral to match these needs. Research on energy storage is continuously growing and is typically motivated by capacity enhancement and energy and/or power density fitting for a specific application. Aside from the performance requirements, the technology must be cost-effective, non-toxic, and sustainable.

**Figure 1:**
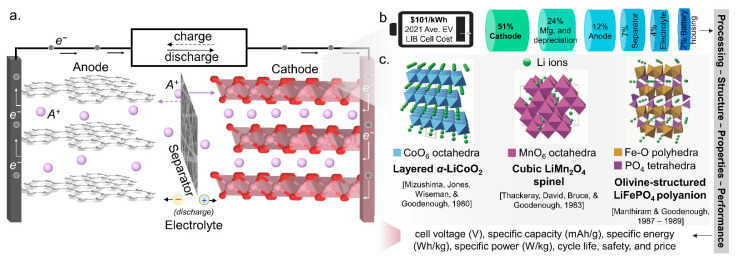
Background and motivation development for secondary battery cathode research: (**a**) schematic diagram of secondary battery principle of operation; (**b**) cost segmentation of electric vehicle lithium-ion battery as of 2021 [3]; and (**c**) conventional cathode materials of the three oxide cathode classifications (Adapted with permission from Ref. [4]. Copyright 2014, The Royal Society of Chemistry). Integral is the foundational understanding of the processing–structure–properties–performance relationship of the cathode materials for the rational design of cost-effective secondary batteries for the intended scale of application.

The general configuration of a “rocking chair” type battery consists of an anode, a cathode, an electrolyte, and a separator (Figure 1a). In the discharge operation of intercalation-type secondary batteries, the anode is oxidized, thereby releasing electrons (*e*^−^) and alkali metal cations (*A^+^*). The *e^–^* flows from the anode to the cathode through a circuit, reducing the cathode. The redox reaction produces an ion current in the electrolyte, controlled by a semi-permeable barrier. Cations move from anode to cathode, while anions move from cathode to anode to maintain a neutral charge on the electrodes. The cation, typically alkali metals (*A^+^*), intercalates in the cathode [2]. The reverse occurs during charging, where *A^+^* ions are deintercalated from the cathode to the anode. 

Among the different battery components, the cathode limits the energy density and influences the battery cost [3,5]. At an average cost of USD 101 kWh^−1^ of electric vehicle lithium-ion battery (LIB) cell in 2021, 51% (~USD 51.5 kWh^−1^) is accounted for by the cathode (Figure 1b) [3]. Hence, the need for a deep understanding of the structure/composition–properties–performance–processing relationship of prospective novel cathode materials to rationally design an effective battery with the required cell voltage, specific capacity, specific energy, specific power, cycle life, safety, and cost [6], among other important parameters, appropriate for the intended scale of application. 

The desirable properties of lithium (Li) [e.g., low relative atomic mass (6.94), low mass-to-electron ratio (6.94), lowest standard potential (−3.04 V), and smallest ionic radius (0.76 Å)] [7] renders the LIB to be the most ubiquitous battery system. This led to the development of the three classes of oxide cathodes by the group of Prof. John Goodenough: (a) layered [8], (b) spinel [9,10], and (c) polyanion oxide cathodes [11,12] (Figure 1c). This development can provide insights into the rational design of cathode materials, especially in the pursuit of a safer [13], more cost-effective [14,15], and equally, if not better performing battery, whether in terms of capacity, lifetime or energy density, than LIB. 

Developing novel cathode materials for next-generation batteries requires considerable efforts involving multidisciplinary approaches. The conventional approach to cathode design employs proven high-performing active materials for LIBs (Figure 1c) or uses compounds containing the mobile cation (e.g., Na, Al, Ca, Mg, or Zn) of the battery chemistry and resemble their discharge products. However, the experimental approach can be time-consuming and expensive given that the chemical space to explore is very large. Hence, computational tools, such as first-principles calculations based on density functional theory (DFT) and machine learning as a subset of Artificial Intelligence, are deemed integral and imperative in discovering novel materials for next-generation batteries [16,17,18].

In one of their works, Ceder et al. [19] reported a series of new mixed polyanion compounds of formula *A*_x_*M*(*Y*O_3_)(*X*O_4_) (*A* = Na, Li; *X* = Si, As, P; *Y* = C, B; *M* = redox-active metal; and x = 0 to 3) identified via high-throughput ab initio computing. The computed stability of both Li- and Na-based compounds was analyzed along with different battery performance parameters such as voltage, specific energy, and energy density of the Li-based compounds. Results suggest that several novel carbonophosphates and carbonosilicates as potential high capacity (>200 mAh g^−1^) and specific energy (>700 Wh kg^−1^) cathode materials for LIBs. Meanwhile, while not as stable as carbonophosphates, borophosphates (BPO) are close enough to stability (<30 meV at^−1^), which motivates further analysis and evaluation of their computed battery properties.

The incorporation of borates can compound the benefit of the tunability and versatility of mixed-polyanion compounds as cathode materials due to their diverse structures and low weight. Borates serve as basic units of trigonal planar BO_3_ and tetrahedral BO_4_, as well as complex polyborate units such as B_2_O_4_, B_2_O_5_, B_3_O_6_, and B_3_O_7_ [20]. This rich chemistry of borates allows a wide range of polyanion ratios in the cathode framework, which can alter the inductive effect, and hence, the cathode potential. Furthermore, borate is generally lighter compared to mature families of polyanion cathode materials such as phosphates [21], silicates [22], and sulfates [23], as the boron element has a lower formula weight than phosphorus, silicon, and sulfur. This translates to a higher theoretical capacity of borate-based compounds relative to other polyanion cathodes. For example, LiFeBO_3_ has a higher theoretical capacity (200 mAh g^−1^) versus the established LiFePO_4_ (170 mAh g^−1^) [24]. This establishes the motivation to investigate further the potential of borate-based cathodes for high-energy density batteries.

This review paper provides an overview first of the development of cathode materials, particularly for LIBs. Understanding this development, motivated mainly by increasing the cell voltage, from the understanding of the electronic structure–properties relationship of elemental components of the conventional LIB cathode materials can provide insights on the rational design of novel cathode materials, particularly of polyanion compounds, for next-generation batteries. The combined data mining and high-throughput ab initio computing methodologies were briefly discussed as to how the carbonate-based compounds were suggested. With the initial recommendation to explore BPO from these methodologies, the limited literature exploring these borate-based compounds, both computationally and experimentally, was consolidated. This review paper then aims to provide a rationale for its further exploration. This also provides a reference on the history, structures, properties, and synthesis routes of known borate-based compounds in the literature. 

## 2. Development and Classifications of Cathode Materials for Secondary Alkali Ion Batteries

Rechargeable (secondary) LIBs were conceived from the advances in intercalation chemistry. The following are general requirements for reversible intercalation reactions: (a) the materials must be crystalline; (b) there must be empty sites in the host crystal lattice in the form of one-dimensional (1D) channels, 2D layers (van der Waals gap), or channels in a 3D network or the form of isolated vacancies; and (c) there must be electronic and ionic conductivity for reversible Li de-/intercalation [25]. Based on these criteria, Brian Steele suggested using transition metal disulfides as intercalation electrode material for secondary LIB at a NATO conference in Italy [26]. The following works evaluated transition-metal dichalcogenide (MS_2_*,* with M = Ta, Nb, and Ti) as electrode materials [27,28,29]. With the fast kinetics of the intercalation reaction in metal disulfides [30,31], Whittingham investigated the electrochemical properties of Li^0^||TiS_2_ battery [32] (Equation (1)) [33] which was then patented in 1975 [34] and 1977 [35]:

xLi + TiS_2_ ⇌ Li_x_TiS*_2_* (1)

The reaction is reversible at room temperature, and the host lattice of the layered compound does not undergo a significant structural change (viz., no phase transition) at the 0 ≤ x ≤ 1 range [33]. The dependence of open-circuit voltage (OCV) on the value of x (i.e., state of charge) is typical for intercalation reactions [33], where most of the intercalation compounds remained single phase only over a relatively narrow range of x values [36]. To maintain reversibility, most of the compounds cannot be cycled in x across the phase transitions of the host material [33]. Table 1 shows examples of early dichalcogenide-based compounds evaluated, and their respective capacities based on reversible range Δx.

Since these dichalcogenide-based compounds were synthesized in the charged state (i.e., x = 0 in Equation (1)), the anode used is Li-metal which serves as the Li source [33]. This results in dendrite growth in the Li-metal anode [37] during cell cycling, which causes internal shorting and presents a fire hazard [5]. In the discharged (lithiated) state, these materials have potential against Li-metal anode, albeit < 3 V and are sensitive to air and water [33]. These constraints on limited OCV, resulting in limited energy density, and the use of dendrite-causing Li-metal anode motivated the increase in battery cell voltage and the development of Li-based cathodes leading to its three classifications: layered oxides, spinel oxides, and polyanion oxides.

### 2.1. Layered Oxides

A larger negative free energy change for a more general reaction (Equation (2))

xA + MX_n_ ⇌ A_x_MX_n_ (2)

is expected when A is small and electropositive, MX_n_ contains a metal atom M at a high oxidation state, and X is small and electronegative [8]. For A, this explains the preference of Li^+^ as intercalating ion. For MX_n_, oxides are preferable over sulfides (e.g., Equation (1)), with oxygen having a smaller ionic radius (1.38 Å vs. 1.84 Å) [38] and being more electronegative (3.44 vs. 2.58) than sulfur [38,39]. Furthermore, a higher oxidation state of M could be more thermodynamically stable as an oxide than as a sulfide [8]. 

**Figure 2:**
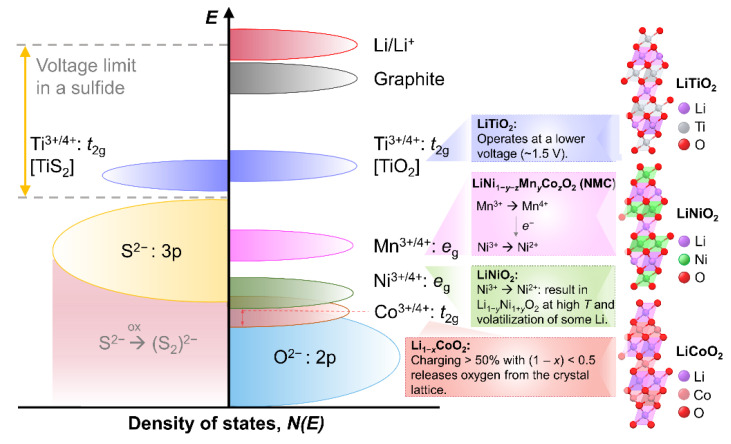
Positions of the redox energies relative to the top of the S^2–^ and O^2–^p bands for the sulfide and oxide cathodes, respectively. Sulfide cathodes have limited cell voltage due to the higher energy of the S^2−^:3p relative to O^2−^:2p (Adapted from Ref. [5]). Sample layered oxide compounds and their respective crystal structures [41] are shown.

The top of the S^2−^:3p band limits the access to lower-lying energy bands with higher oxidation states (e.g., Co^3+/4+^) and hence, higher cell voltage [5]. Lowering of cathode redox energy via access to higher oxidation states in sulfide results in the oxidation of S^2−^ ions to molecular disulfide ions (S_2_)^2−^ (Figure 2) [5]. Meanwhile, the top of the O^2−^:2p band lies at lower energy which allows the significant reduction in the redox energy of oxide-based cathodes via access to lower-lying energy bands (e.g., Co^3+/4+^). This rationale by Prof. John B. Goodenough [40] led to the investigation of the first oxide cathode, layered Li_x_CoO_2_ (0 < x ≤ 1) [8].

**Figure 3:**
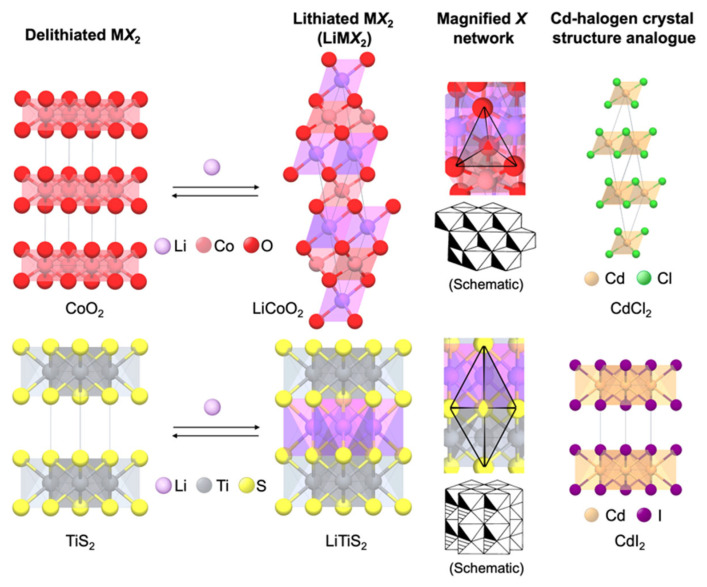
Comparison of the crystal structures [41] of the delithiated and lithiated CoO_2_ and TiS_2_, which are analogous to the crystal structures of the close-packed pseudocubic array of CdCl_2_ and close-packed hexagonal array CdI_2_, respectively.

In their work, Mizushima et al. [8] evaluated whether Li-ion mobilities in layered, metallic oxides can be high enough to sustain large voltages reversibly at current densities of the order 1 mA cm^−2^. The Li^+^ and M^3+^ ions in LiMO_2_ occupy alternate [111] layers of the rocksalt structure; delithiation will yield an MO_2_ phase with the CdCl_2_ structure (Figure 3). The main difference between the CdCl_2_ and the CdI_2_ structure of TiS_2_ is in the close-packed arrangement of the anions: anions form a close-packed pseudocubic array in CdCl_2_ while they form a close-packed hexagonal array in CdI_2_ (Figure 3). Their findings show that overvoltage and reversibility are feasible, at least to x = 0.5 and to current densities of 1 mA cm^−2^. This could be due to the high Li-ion mobility relative to preliminary data on other LiMO_2_ (M = V [42], Cr [43], Co [44], and Ni [45]) compounds. This can be attributed to the following: (1) the spacing between oxygen layers facing the Li layer is greater in LiCoO_2_ than in other LiMO_2_; (2) the high electron affinity of the low-spin Co^3+/4+^ couple makes the oxygen layers strongly polarizable toward the cobalt layer; and (3) X-ray powder refinements indicate the almost complete ordering of Li-Co into alternate [111] layers which is not true especially to the corresponding Ni compound [8]. This high ionic diffusivity and reversibility of Li (minimum at x = 0.067) in layered Li_x_CoO_2_ renders the Li_x_CoO_2_||Li cells with high OCV of 4–5 V allowing the window for evaluating alternate non-Li anodes. At a compositional range of 0.067 ≤ x ≤ 1, the Li_x_CoO_2_ layered structure was kinetically stable at room temperature in the electrolyte (i.e., 1 M LiBF_4_ solution in propylene carbonate soaked onto Whatman GF/D glass-fiber paper) [8].

Despite the promising electrochemical performance of LiCoO_2_, charging to more than 50% (1 − x < 0.5) in the Li_1-x_CoO_2_ cathode leads to a release of oxygen from the crystal lattice [46,47], which could be due to the overlapping Co^3+/4+^ band with the top of the O^2−^:2p band (Figure 2). This results in the limited practical capacity of LiCoO_2_ at ~140 mAh g^−1^.

Several layered LiMO_2_ (M = 3*d* transition metals) have been investigated following LiCoO_2_ and other pioneering works [42,43,44,45]. LiTiO_2_, aside from its tedious synthesis at lower-valent Ti^3+^, operates at a lower voltage (~1.5 V) (Figure 2), making it not a suitable cathode material [5]. LiNiO_2_ is also limited by a difficult synthesis route, as Ni^3+^ tends to be reduced to Ni^2+^ [5]. This results in Li_1−y_Ni_1+y_O_2_ at high-temperature synthesis (~700–800 °C), with some Li being volatilized [48,49]. LiMnO_2_, along with M = V and Fe, experiences layered to spinel transitions or other structural changes during charge–discharge due to low octahedral-site stabilization energy (OSSE) [5,50,51], thus limiting it to be a good cathode material. The limited capacity (~140 mAh g^−1^) of LiCoO_2_ and the high cost of Co motivated its substitution with Mn and Ni, which yields the LiNi_1−y−z_Mn_y_Co_z_O_2_ (NMC) material.

The properties and performance of these layered ternary cathodes are a function of the relative amounts of the transition metals (Ni, Co, Mn) [52]. Increased Ni content improves the discharge capacity and potential of NCM LIBs as a function of Ni oxidation states (Ni^2+^/Ni^3+^ and Ni^3+^/Ni^4+^) while decreasing the cyclability and thermal stability of the LIB [53] due to the chemical and structural instabilities of Ni^4+^ at the charged state [52]. Co, while expensive, improves the rate performance and conductivity mainly due to the exhibited itinerant electron system of Co in the LiNi_x_Co_y_Mn_z_O_2_ cathode [54]. Meanwhile, Mn present as Mn^4+^ reduces Ni^3+^ to Ni^2+^, causing Li^+^/Ni^2+^ mixing. This forms a compact structure within the layered crystalline symmetry which eliminates further disorder in cycling, thereby improving the cycling stability of Mn-doped high-Ni layered oxide cathodes LiNi_0.9_Mn_0.1_O_2_ [53]. To reduce cost and increase energy density, compositions with low-Co/high-Ni content are targeted. LiNi_0.65_Co_0.15_Mn_0.2_O_2_ performed at a relatively high capacity (186.5 mAh g^−1^, at 0.1 C-rate, 2.8–4.3 V) with 80.3% capacity retention (900 cycles at 60 °C) [52] and nanorod LiNi_0.6_Co_0.2_Mn_0.2_O_2_ exhibited a capacity of 152.2 mAh g^−1^ at 5 C-rate with 90.6% capacity retention (200 cycles) [55]. These compounds and their modification strategies are extensively reviewed elsewhere [56,57].

### 2.2. Spinel Oxides

Propelled by the severe corrosion issue encountered among Li-transition metal sulfide systems, Thackeray et al. [9,58] investigated the behavior of iron oxide cathodes (viz., *α*-Fe_2_O_3_ and Fe_3_O_4_). Although the seminal work regarded the iron oxides as potential cathode candidates for high energy-density batteries, the *α*-Fe_2_O_3_ and Fe_3_O_4_ had limited OCV of 1.86 V and 1.81, respectively [58]. Nevertheless, the successful demonstration of Li insertion into the spinel Fe_3_O_4_ provided the following relevant information from the structural and electrochemical data on lithiated Fe_3_O_4_, Li_x_Fe_3_O_4_: (1) Li electrochemical insertion can have a wide compositional range at 0 ≤ x ≤ 2; (2) at x = 1.3 and exposure on air, Li_x_Fe_3_O_4_ tends to burn suggesting that some of the Li in excess of x = 1 diffuses to the surface and is readily oxidized; and (3) at 0 ≤ x ≤ 1, Li enters the 16*c* positions and displaces the Fe^3+^ on 8*a* sites into other 16*c* positions, but the [B_2_]X_4_ framework of the spinel structures remains intact [9]. This motivated the investigation of the electrochemical extraction of Li from LiMn_2_O_4_ with A[B_2_]X_4_ framework [10,59].

**Figure 4:**
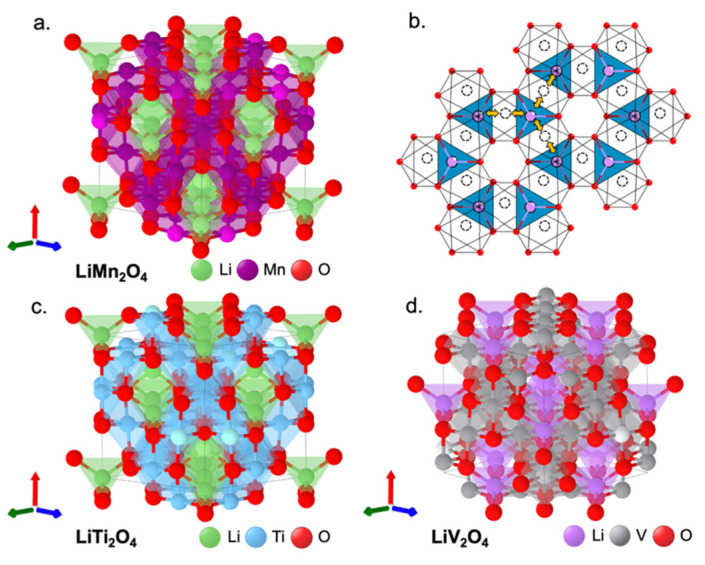
Crystal structures (cubic spinel characterized by space group *Fd*$\bar{3}$*m*) of representative A[B_2_]X_4_ framework [41]: (**a**) LiMn_2_O_4_; (**c**) LiTi_2_O_4_; and (**d**) LiV_2_O_4_ and (**b**) Li-ion diffusion in the framework (Adapted from Ref. [5]).

In a cubic spinel characterized by space group *Fd*$\bar{3}$*m*, A and B are the cations in tetrahedral (8*a*) and octahedral (16*d*) sites, respectively. Atoms X are cubic-close-packed anions (32*e*). For LiMn_2_O_4_, Li^+^ occupies the 8*a* tetrahedral sites, while Mn^3+/4+^ occupies the 16*d* octahedral sites (Figure 4a).

The Li-ion diffusion occurs from one 8*a* tetrahedral site to another 8*a* tetrahedral site via a neighboring empty 16*c* octahedral site (Figure 4b) as it has the lowest energy barrier [5,10]. This edge-shared octahedra in the spinel cathode (e.g., [Mn_2_]O_4_) offers a three-dimensional Li-ion diffusion pathway with fast Li-ion conductivity [10,59]. This renders the Li_1−x_Mn_2_O_4_ with faster charge–discharge characteristics with good reversibility compared to layered LiCoO_2_ with only two-dimensional Li-ion diffusion. The de-/intercalation of Li from/into the tetrahedral sites in Li_1−x_Mn_2_O_4_ offers a high operating voltage of 4 V with a practical capacity of <130 mAh g^−1^ as close to one Li per two Mn ions can be reversibly extracted from the tetrahedral sites [5]. 

The electrochemical curves for the Li removal from Li[Mn_2_]O_4_ indicate an immediate sharp drop followed by a gradual decline in voltage which indicates the formation of a single-phase Li_1−x_[Mn_2_]O_4_ [59]. At a current density of 15 μA cm^−2^, x ≈ 0.6 could be withdrawn from the spinel. For x > 0.6, the OCV versus composition curve suggests the onset of a different electrochemical process. At a current density of 15 μA cm^−2^, x ≈ 0.5 before the onset of the second electrochemical process. This electrochemical process was hypothesized to be due to the exhaustion of Li-ion at the particle surface, which could proceed to the production of λ-MnO_2_ at a smaller particle size and a lower current density [59]. This was investigated and confirmed by comparing the XRD spectra of acid-treated LiMn_2_O_4_ and electrochemically delithiated Li_1−x_[Mn_2_]O_4_ at x > 0.6 where disproportionation reaction (Equation (3)) was deduced [59,60] in the presence of H^+^ in the electrolyte:

2Li[Mn_2_]O_4_ + 4H^+^ (aq) → 2Li^+^ (aq) + Mn^2+^ (aq) + H_2_O (l) + 3λ-MnO_2_ (3)

In this disproportionation, Mn^4+^ is retained in the solid while Mn^2+^ is leached out into the electrolyte, resulting in cathode degradation and (graphitic) anode poisoning, thereby limiting the cycle life of LIBs [61]. Alteration of the long-range Mn-Mn interaction via trace Li substitution (e.g., 5 atom%) of Mn in LiMnO_4_ alleviates the Mn^3+^ disproportionation reaction, thereby reducing Mn dissolution and consequently improving cyclability [5].

Due to the difficulty in stabilizing highly oxidized M (e.g., M^3+/4+^) by conventional high-temperature synthesis, spinel Li[M_2_]O_4_ are known only with M = Mn, Ti, and V (Figure 4a,c,d). However, LiTi_2_O_4_ and LiV_2_O_4_ are limited with low OCV at 1.5 V [5] and 3 V [62], respectively. Similar to the abatement of Mn dissolution, partial substitution of Mn with other ions such as Cr, Co, and Ni has been explored to improve these limited performances. With Ni^2+/3+^ and Ni^3+/4+^ couples (as in NMC cathodes) and tetrahedral-site Li-ions in Ni-substituted Mn, spinel LiMn_1.5_Ni_0.5_O_4_ (LMNO) [63,64] was found to operate at ~4.7 V with a reversible capacity of ~135 mAh g^−1^. However, a suitable electrolyte that can be stable at such high voltages is lacking, resulting in the capacity fade of LMNO [65]. 

### 2.3. Polyanion Oxides

By the late 1990s, Li-intercalating compounds, viz*.,* layered Li_1−x_CoO_2_ and spinel Li_1−x_[Mn_2_]O_4_, were already commercialized as 4 V cathode materials for secondary LIBs. However, they are still restricted with metastability: Li_1−x_CoO_2_ loses O_2_ at *T* > 180 °C [66], while [Mn_2_]O_4_ converts to *ε*-MnO_2_ at 190 °C [67]. Furthermore, Jahn-Teller deformation of the spinel Li_1−x_[Mn_2_]O_4_ irreversibly reduces the capacity for its repeated cycling [68]. These, aside from the availability and cost issues of transition metals, motivated the investigations on iron-based oxides. 

**Figure 5:**
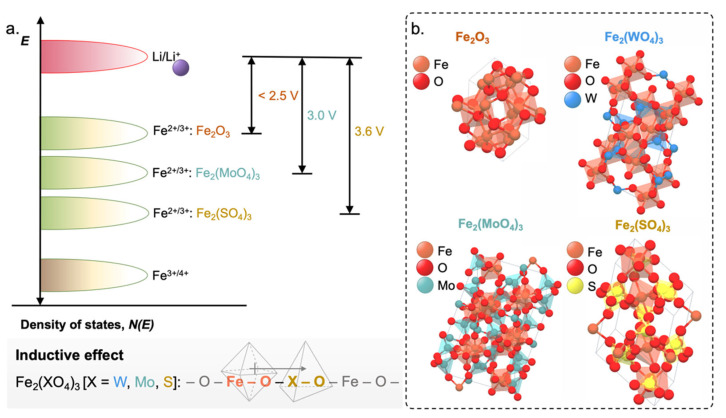
Rationale for polyanion compounds as cathode materials: (**a**) role of counter-cations in altering the redox energies in polyanion oxides as exhibited by inductive effect–polarization of the O^2−^ electrons into the strong covalent bonding within the polyanion reduces the covalent bonding to the Fe-ion which consequently lowers its redox energy (Adapted from [5]); (**b**) crystal structures [41] of simple oxide Fe_2_O_3_ and Fe-polyanion oxides.

The redox energies of Fe species in Fe-based oxides pose limitations to its performance with respect to the Li anode. The Fe^3+/4+^ redox energy lies too distant below the Fermi energy of a Li anode and is beyond the electrochemical window of the electrolyte (Figure 5a) [69,70]. Meanwhile, Fe^2+/3+^ redox energy is too close to that of Li/Li^+^, which results in a too low voltage of the cell (Figure 5a). This behavior could be related to the strong interactions among the *d* electrons and the high spin configuration of Fe^3+^ [70]. Meanwhile, employment of polyanions such as (PO_4_)^3−^, (SO_4_)^2−^, (AsO_4_)^3−^, (MoO_4_)^3−^, or (WO_4_)^2−^ can lower the Fe^2+/3+^ redox energy to useful levels (Figure 5a) [69]. For example, Fe_2_(MoO_4_)_3_ and Fe_2_(WO_4_)_3_ exhibited a flat discharge voltage of 3 V [9] while Fe_2_(SO_4_)_3_ displayed much higher at 3.6 V [12]. These are significantly higher compared to Fe_2_O_3_ or Fe_3_O_4_, which had flat discharge voltage <2.5 V operating with the same Fe^2+/3+^ redox couple (Figure 5a). This trend revealed the effect of counter cations (viz., Mo^6+^, W^6+^, and S^6+^) in shifting the redox energy of the Fe^2+/3+^. There is a reduction in Fe^2+/3+^ redox energy and an increase in the OCV versus Li, with increasing strength of covalent bonding within the polyanion. Polarization of the O^2−^ electrons into the strong covalent bonding within the polyanion reduces the bond strength towards the Fe-ion, which consequently lowers its redox energy [69]. Structurally, the Fe_2_(XO_4_)_3_ (X = Mo, W, and S) structure shows that the FeO_6_ octahedron share its corners with the XO_4_ tetrahedra (Figure 5b). This provides a three-dimensional –O–Fe–O–X–O–Fe–O– extended linkage. Consequently, the Fe–O bond strength is weakened by the inductive effect of the increased strength of the X–O bond. This results in the lowering of the Fe^2+/3+^ redox energy and a corresponding increase in the operating voltage with respect to the XO_4_-free Fe_2_O_3_ (Figure 5a). 

These studies on polyanion-based cathodes employing molybdate [11], sulfate [12], and phosphate [71] and the inductive effect [11,12] motivated the study of Padhi et al. [69] on new series of compounds viz., LiMPO_4_ (M = Fe, Mn, Co, or Ni), which has the ordered olivine structure. In this study, LiMnPO_4_, LiCoPO_4_, and LiNiPO_4_ were unsuccessful in delithiation using LiClO_4_ as an electrolyte. Meanwhile, the deintercalation (Equation (4))—intercalation (Equation (5)) of Li-ions into the LiFePO_4_ was not only reversible on repeated cycling, but the capacity was also observed to be slightly increasing with cycling [69].

LiFePO_4_ − xLi^+^ − x*e*^−^→ xFePO_4_ + (1 − x)LiFePO_4_ (4)

FePO_4_ + xLi^+^ + x*e*^−^ → xLiFePO_4_ + (1 − *x*)LiFePO_4_ (5)

**Figure 6:**
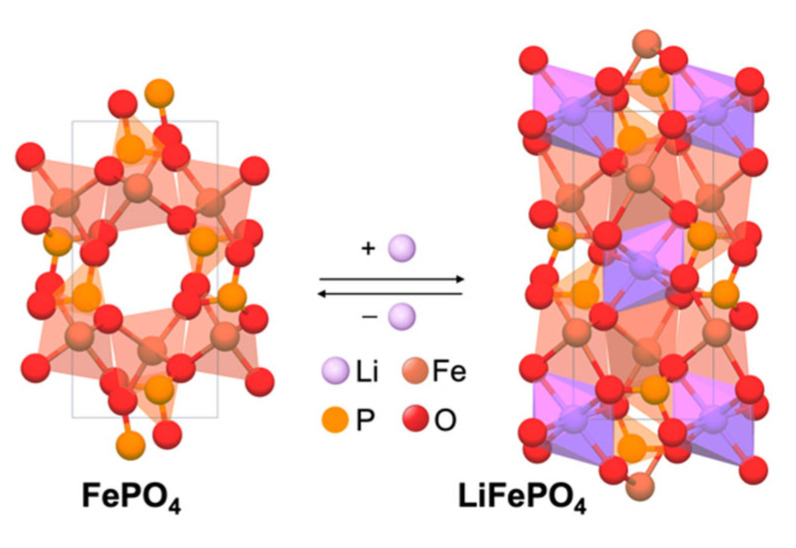
Crystal structure [41] and schematic diagram of the Li-ion de-/intercalation in olivine LiFePO_4_.

The V(x) curves of Li_1−x_FePO_4_ exhibit a voltage that is independent of over a large range of x. This indicates that the Li de-/intercalation proceeds by the motion of a two-phase interface based on Gibb’s phase rule, where the second phase was elucidated to be FePO_4_ (Equations (4) and (5)) via XRD analyses [69]. The excellent reversibility of the cells on repeated cycling is mainly due to the similarity of the LiFePO_4_ and FePO_4_ structures (Figure 6) [69].

The good Li-ion de-/intercalation reversibility and the employment of inexpensive, abundant, and environmentally benign elements render the LiFePO_4_ a highly promising cathode material. However, it only supports relatively small current densities at room temperature. The nearly close-packed hexagonal oxide-iron array is strongly bonded in three dimensions and provides a relatively small free volume for Li-ion motion. While increasing the current density decreases the cell capacity, this decrease is reversible. That is, reducing the current restores the capacity. Furthermore, increasing the current density does not lower the OCV [69]. This indicates that the loss in capacity in LiFePO_4_ is a diffusion-limited phenomenon associated with the two-phase character of the intercalation process (Figure 6). 

The positive result of inductive effect (Figure 5) on the metal–oxygen bonding in tuning the operating voltages renders the polyanion-based cathode (e.g., LiMPO_4_) with voltage as high as ~5 V even with lower-valent couples such as Co^2+/3+^ or Ni^2+/3+^ [5,72]. Despite the smaller theoretical gravimetric capacity due to the presence of polyanion groups such as SO_4_^2−^, PO_4_^3−^, and SiO_4_^4−^, the stable frameworks and immense variety of atomic arrangements and crystal structures render the polyanionic frameworks as cathode materials for secondary Li or Na batteries [72].

### 2.4. Advantages and Disadvantages of Oxide Cathodes

The three classes of oxide cathodes possess different properties (Table 2) and electrochemical activities (Table 3) as a function of their composition and structure which render them with their respective advantages and disadvantages. Both layered and spinel oxides have close-packed structures with high densities. Meanwhile, polyanion oxides have lower densities, given the porous nature of the structure. Both the layered and spinel class of oxides offer good electronic conductivity, while polyanion oxides are poor electrical conductors. To increase conductivity, polyanion oxide cathodes require a particle size reduction and a conductive carbon coating. This often increases the processing cost and introduces inconsistencies in performance. The necessity for small particles also further reduces polyanion cathode densities, leading to a lower volumetric energy density. Thus, polyanion cathodes are less attractive for applications that require high volumetric energy density, such as portable electronic devices and electric vehicles, compared to layered oxide cathodes [5].

On the other hand, polyanion cathodes with optimally small carbon-coated particles can sustain high charge–discharge rates due to good structural integrity, despite a lower volumetric energy density. Moreover, polyanion cathodes are known to form compositions with earth-abundant transition metals such as Fe, unlike the layered and spinel oxides, offering sustainability advantages. Therefore, polyanion oxides are appealing for stationary grid storage of electricity produced from renewable energy sources such as solar and wind [5], where weight is not a primary consideration.

In terms of electrochemical activity (Table 3), layered oxides offer relatively high cell voltages (~3.5 V) with high theoretical specific capacity (~275 mAh g^−1^). Spinel oxides offer intermediate voltage (~2.5 V) and specific capacity (~150 mAh g^−1^) relative to the other two classes. The polyanion class offers higher cell voltages (> 3.5 V) as a result of the inductive effect at the expense of relatively lower specific capacities (~150 mAh g^−1^) due to their low density. Furthermore, polyanion cathodes offer an important advantage in terms of high thermal stability and better safety compared to layered and spinel oxide cathodes. Better stability in polyanion cathodes can be attributed to oxygen tightly bound to P, S, or Si through covalent bonds in the polyanion unit. These different factors provide good targets for rational design to improve the properties of currently available battery chemistries, such as specific energy, energy density, power density and safety. 

## 3. Combined Data Mining and High-Throughput ab Initio Computing Methodology for Novel Cathode Materials Development

**Figure 7:**
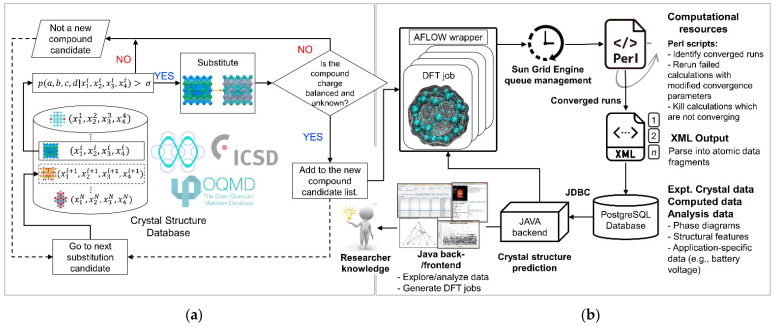
Schematic diagram of Ceder et al. combined data mining and high-throughput ab initio computing methodology for novel cathode materials development: (**a**) substitutional probabilistic model to predict new compounds formed by a, b, c, and d species (Adapted with permission from Ref. [93]. Copyright 2011, American Chemical Society); (**b**) data flow implementation in their high-throughput project (Adapted with permission from [102]. Copyright 2011, Elsevier).

To propose a novel cathode material, Hautier et al. [19] employ a combination of data mining (Figure 7a) with DFT (Figure 7b). Starting with known compounds present in a crystal structure database, their group performs a series of chemical substitutions. Each compound containing $\left(x_{i}^{1},\;x_{i}^{2},\;x_{i}^{3},\;x_{i}^{4}\right)$ as ionic species, the $p\left(a,\;b,\;c,\;d|x_{i}^{1},\;x_{i}^{2},\;x_{i}^{3},\;x_{i}^{4}\right)$ is calculated to evaluate the probability of forming a new compound via substitution of $x_{i}^{1},\;x_{i}^{2},\;x_{i}^{3},\;and\;x_{i}^{4}$ with a, b, c, and d. At a probability higher than a given threshold σ, the substituted structure is considered and added to the group’s list of new compounds, given that the candidate compound is charge balanced and previously unknown [93]. The selected data of the candidate compound, which are Java-coded [94,95,96], are then employed for DFT jobs, wrapped by the Automatic FLOW (AFLOW) [97] using Java back end (Figure 7b). The batches of DFT jobs are submitted to a Grid Engine queuing system [98], where active jobs are monitored and converged using Perl scripts [99]. Completed DFT jobs are inputted into a PostgreSQL [100,101] database, which interfaces with the Java back end through Java Database Connectivity (JDBC). Herein, a graphical front end allows for data exploration and analysis.

The cathode material candidate Li_3_Mn(CO_3_)(PO_4_) generated through a Li to Na substitution on the known material sidorenkite, Na_3_Mn(CO_3_)(PO_4_) [103] was identified by Ceder et al. through their high-throughput computational search [19]. The sidorenkite structure is composed of distorted manganese octahedra connected to four PO_4_ groups on its vertices. Each Mn octahedron is also connected to a carbonate group [104]. The sidorenkite crystal structure, which has up to three alkali metals per redox metal, can theoretically render multiple electron activity during delithiation when combined with an adequate redox couple [19,105]. For example, Li_3_Mn(CO_3_)(PO_4_) has a theoretical capacity of up to 232 mAh g^−1^ (versus the commercially used polyanionic cathode LiFePO_4_, which has a capacity of ~170 mAh g^−1^). With this promising high capacity, different polyanionic mixtures in the sidorenkite structure were explored by computing total energies (and hence, the thermodynamic stability) for all combinations of *A*_x_*M*(*Y*O_3_)(*X*O_4_) formula with *A* = Li, Na; *X* = Si, As, P; *Y* = C, B; *M* = a redox-active metal; and x = 0 to 3.

From the high-throughput ab initio computation search, the sidorenkite crystal structure favors Na on the alkali site, and none of the Li-based compounds were found to be thermodynamically stable. However, Li-containing compounds for LIB could be synthesized by first forming the stable Na compound and performing a Li- to Na-ion exchange to produce the metastable Li-containing compound, which is not uncommon in battery material synthesis [51,106,107]. Given the evaluated intrinsic electrochemical properties such as delithiation voltages, stability of the Na form, voltages compatible with commercial electrolytes, specific energy, and energy densities, manganese [Li_3_Mn(CO_3_)(PO_4_)] and vanadium [Li_2_V(CO_3_)(PO_4_)] carbonophosphates, and the vanadium [Li_3_V(CO_3_)(SiO_4_)] and molybdenum carbonosilicates [Li_3_Mo(CO_3_)(SiO_4_)] were found to be promising (Table 4).

Meanwhile, while not as stable as carbonophosphates and carbonosilicates, borate-based compounds are close enough to stability (viz., <30 meV at^−1^), which motivated the analysis of its battery properties (Table 4). Results show that if these could be synthesized, borate-based compounds, especially BPOs, would have very promising specific energy and energy density in electrolyte stability. This intensifies the novelty and motivation of the naturally existing borate-based compounds [108] for the search and development of new cathode materials with better specific energy, energy and power density, safety, and economic feasibility.

## 4. Borate-Based Compounds as Cathodes

Empty sites in the host crystal lattice in the form of 1D, 2D, or channels in a 3D network or in the form of isolated vacancies can provide good ion diffusivity required for effective secondary batteries [25]. Because of their structural stability, borate-based compounds are excellent candidates for cathode materials, as evidenced by the lack of phase transition during the charging/discharging process. A very small volume change for Li_x_FeBO_3_ (−2%, 0.15 < x < 1) was observed compared to the typical intercalation compounds Li_x_FePO_4_ (−6.5%, 0 < x < 1), Li_x_MnPO_4_ (−10%, 0 < x < 1) and Li_x_Mn_2_O_4_ (−7.3%, 0 < x < 1) [24]. Iron borophosphate, Li_0.8_Fe(H_2_O)_2_[BP_2_O_8_]·H_2_O (LiFe-BPO) [109], was also shown to be highly crystalline and that the structural framework of the delithiated phase preserved that of the parent lithiated phase supporting a solid-solution mechanism [109]. This proffers borate-mixed polyanion compounds as it can exhibit various 2D or 3D structures (e.g., layered, helical, chain, ribbon, and channel forming open framework structures), theoretically allowing Li- or Na-ion intercalation. 

Computationally, Ceder et al. [19] predicted the electrochemical properties of borate-based mixed polyanion compounds in a sidorenkite structure. Comparing these computed properties (Table 4) with that of commercial oxide cathodes such as layered LiCoO_2,_ LiMnO_2_, spinel LiMn_2_O_4_, LiV_2_O_4_, and polyanion LiFePO_4_, LiCoPO_4_, LiMnPO_4_ (Table 3), the borate-based cathodes have higher voltages and specific capacities that are competitive, if not higher. The intercalation-favorable structures and the theoretical performance advantages of borate-based compounds motivate their evaluation as cathode materials. This review paper focuses on the known borate-based compounds [viz., borophosphate (BPO), borosilicate (BSiO), and borosulfate (BSO)]. 

**Figure 8:**
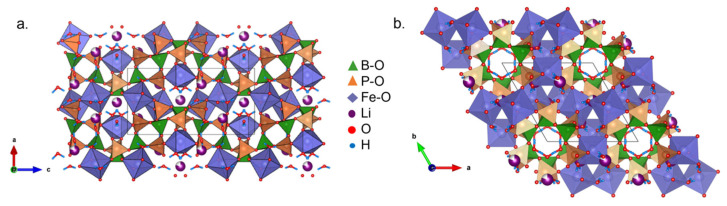
Structure of the LiFe-BPO cathode [109]. The polyhedral connectivity of LiFe-BPO viewed along the (**a**) b-axis and (**b**) c-axis. The [∞1(B2P4Φ16)_m_] (*Φ* = O, OH) helical chains form a channel structure filled with free water molecules. The channels are interconnected by FeO_6_ polyhedra, forming gaps in the structure that host the Li^+^ ion.

Alkali-ion intercalation in BPO cathodes has been previously reported. Li- and Na-ion intercalation were investigated in Li_0.8_Fe(H_2_O)_2_[BP_2_O_8_]·H_2_O (LiFe-BPO) [109], while Na-ion intercalation [110] was studied in previously synthesized (NH_4_)_0.75_Fe(H_2_O)_2_[BP_2_O_8_]∙0.25H_2_O (NHFe-BPO) [111] and NaFe(H_2_O)_2_[BP_2_O_8_]·H_2_O (NaFe-BPO) [112]. The studied BPO cathodes have similar structures (Figure 8). They are composed of BO_4_ and PO_4_ tetrahedra that form helical [∞1(B2P4Φ16)_m_] (*Φ* = O, OH) chains (Figure 8a). This results in a tube structure joined together by octahedrally coordinated FeO_6_. The tunnel-like structures host free H_2_O molecules (Figure 8b)_._ Pores created by FeO_6_ can host the alkali metal ions. The tube structure and the presence of redox-active Fe in the BPO provide the potential for alkali metal intercalation required for secondary battery cathode application.

Li- or Na-ion intercalation was successfully demonstrated on the Fe-BPO cathodes, albeit the inferior performance with respect to commercial batteries. Cathode potentials were ~3 V while the specific capacities ranged from 66 to 80 mAh g^−1^ (Table 5) versus the cathode potential of ~3.5 V and specific capacity of 150–200 mAh g^−1^ of commercial batteries. For the same cathode, LiFe-BPO, the theoretical specific capacity of the Na-ion cell is lower than that of the Li-ion cell (80.33 vs. 84.4 mAh g^−1^). A broad anodic peak and peak position shifts were observed in the cyclic voltammetry (CV) curves of the Na-ion cells, implying a higher degree of polarization and lower redox stability than the latter. Upon cycling, the Na-ion cell experienced ~9% capacity loss after the third cycle, indicating a lower degree of reversibility compared to the Li-ion cell. This can be due to the increased ionic diffusion energy barrier introduced by Na-ion’s larger size than Li^+^, which limits its mobility in the BPO structure, as corroborated by a more pronounced capacity degradation of the LiFe-BPO/Na cell and an impedance of more than three times that of the LiFe-BPO/Li cell [109]. Likewise, the delivered capacity of the NaFe-BPO and NHFe-BPO cathodes dropped drastically at the 10th and 40th cycles, respectively. This could be attributed to electrolyte degradation brought on by irreversible side reactions during oxidation [110]. These results also suggest the effect on the performance of a particular battery chemistry is based on the selectivity of the cathode material on the intercalating ion and electrolyte. 

A few BSiOs were identified to be close to stability by Ceder et al. (Table 4) [19]. For example, Li-exchanged Na compounds of Li_3_Mo(BO_3_)(SiO_4_), Li_3_V(BO_3_)(SiO_4_), and Li_3_Bi(BO_3_)(SiO_4_) are within 30 meV at^−1^. This metastability of BSiO could have led to the limited literature on BSiO as cathode material. Meanwhile, orthosilicate compounds with the formula Li_2_MSiO_4_ (M = Mn, Fe, and Co) have been investigated [113] as analogues of commercial LiFePO_4_, as P and Si are adjacent elements. Orthosilicates can provide higher lattice stabilization due to the stronger Si-O than the P-O bond [89,113]. However, they can be limited by low electronic conductivity (~10^−12^ S cm^−1^) [114,115,116] and Li^+^ diffusion coefficient [117]. To improve the current rate discharge, LiFeSiO_4_ was co-doped with a metal ion (e.g., Mg^2+^ and Ag^+^) in the Fe site and borate in the Si site [117,118]. The introduced defects can favor the Li-ion de-/intercalation. Both were synthesized via a solid-state reaction and were observed to have a monoclinic structure (*P*2_1_/*n*) (Table 6). 

Li_2_Fe_0.98_Mg_0.02_(SiO_4_)_0.97_(BO_3_)_0.03_/C [117] and Li_2_Fe_0.98_Ag_0.02_(SiO_4_)_0.99_(BO_3_)_0.01_/C [118] were shown to have good electrochemical performance (Table 6). Li_2_Fe_0.98_Mg_0.02_(SiO_4_)_0.97_(BO_3_)_0.03_/C was reported to have good cyclic stability and an initial discharge capacity of 138 mAh g^−1^ while Li_2_Fe_0.98_Ag_0.02_(SiO_4_)_0.99_(BO_3_)_0.01_/C had 150.8 mAh g^−1^ with a capacity retention of 87% after 10 cycles at 0.1 C-rate. The reported compounds had relatively low charge transfer impedance values compared to other LiFeSiO_4_ compounds prepared at different doping ratios. However, they had poor charge–discharge reversibility based on CV. These works can provide insights into the challenges and opportunities for BSiO-based cathode materials.

Ceder et al. reported that sulfate-based chemistries are by far the most unstable in the sidorenkite crystal structure, which includes BSOs which are higher than 30 meV at^−1^ above the convex hull [19]. This could explain the lack of literature on BSO-based cathode. Nevertheless, BSOs are structurally analogous to existing polyanionic cathodes, which have gaps or channels [119,120,121] which can provide pathways for cation de-/intercalation during charging and discharging. The corner-sharing tetrahedra topology of BSOs is proven to be stable according to Pauling’s rule [122].

The multiple atomic compositions of polyanions which allows versatile engineering, the rich chemistry and diverse molecular architecture of borate mixed polyanions which can provide the site for alkali ion intercalation, and the untapped potential of these borate-based compounds prompt its review and investigation. Its abundance and hence, cost advantage can also compound its capacity as a cost-effective and high-performing cathode material for secondary batteries. The succeeding sections further discuss the structures, properties, and synthesis routes of identified potential borate-based cathode materials, BPO and BSiO. BSO, despite its instability in the sidorenkite crystal structure, was also included for engineering purposes.

## 5. Borophosphates (BPO)

BPO, particularly in the sidorenkite structure, has promising specific energy and energy density (Table 4) [19]. Li- and Na-ion intercalation in the isostructural LiFe-BPO [109], NHFe-BPO, and NaFe-BPO [110] further suggest their potential as cathode materials. 

BPO contains complex anionic partial structures and has been explored because of their unique physicochemical properties [123]. One of the earliest reported phases of BPOs was M[BPO_5_] (M = Ca, Sr) in 1965 [124]. The compound Ca[BPO_5_] was confirmed via X-ray powder diffraction and optical microscopy in 1974 [125]. BPOs were found to have a similar crystalline structure with stillwellite, CeBSiO_5_, with helical chains of corner-sharing BO_4_ tetrahedra [126]. The structures, properties and synthesis routes of known and synthesized BPO compounds in the literature are quite diverse and are presented in the paragraphs that follow. 

### 5.1. Structures

BPOs have various open frameworks constructed from borate and phosphate units that condense in different structural motifs. They are considered silicate-analogues as they contain similar building blocks to SiO_4_ where instead of silicon, boron and phosphorus occupy the tetrahedral center. In addition, borates can either be three- or four-oxygen coordinated, increasing the structural diversity of BPOs. Likewise, BPO structures were initially described similarly to silicates [127] which are based on the linking between SiO_4_ building block units [128]. This description was refined to account for the complex structural combinations of borates and phosphates [129]: (1) anhydrous or hydrated phases; (2) based on boron-to-phosphorous (B:P) molar ratio and degree of protonation [127]; (3) structural parameters (e.g., connectedness, branchedness, dimensionality, and periodicity have been established) [127,128]. A large part of the structural diversity of BPOs is due to the B:P ratio and avoidance of the P-O-P connection. A proposed explanation for the lack of P-O-P bonds is based on Pauling’s fourth rule [130], stating that for crystals with different cations, those with a high charge and low coordination number have the tendency to avoid sharing polyhedra. This classification by coordination number initially distinguishes the BPOs.

**Figure 9:**
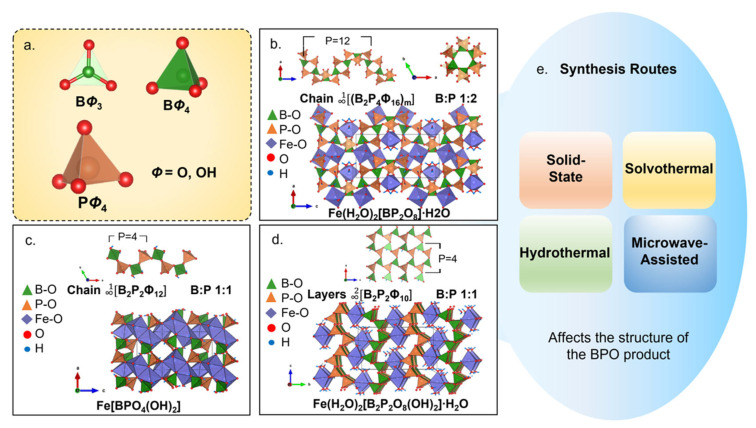
(**a**) The construction of BPO structures consists of at least one borate unit, three- or four-coordinated, BΦ_4_ and one phosphate unit, PΦ_4_. (**b**) Fe(H_2_O)_2_[BP_2_O_8_]∙H_2_O (bottom) [131] with its BPO 1D helical chain FBU structure (top). (**c**) Fe[BPO_4_(OH)_2_] (bottom) [132] with its BPO 1D chain FBU structure (top). (**d**) Fe(H_2_O)_2_[B_2_P_2_(OH)_2_]∙H_2_O (bottom) [133] with its BPO 2D 6^3^ net-topology FBU structure (top) (FBUs were adapted with permission from [130]. Copyright 2007, WILEY-VCH Verlag GmbH & Co. KGaA, Weinheim). (**e**) Synthesis routes of BPO compounds.

BPOs are constructed from trigonal-planar BΦ_3_ and/or tetrahedral BΦ_4_ and tetrahedral PΦ_4_, where Φ = O, OH (Figure 9a) [130]. Borate and phosphate polyhedra share common corners to form basic building block units (BBU) that condense into representative oligomers called fundamental building units (FBU). The FBUs contain the essential structural pattern of the BPO. The FBUs vary based on their B:P ratio and on the linking between borates and phosphates. Thus, some B:P ratios are favored over others causing particular motifs to be observed more frequently, such as open-branched chains, three-, four-, and six-membered rings, and B:P = 1:2 helical chains. For example, Fe(H_2_O)[BP_2_O_8_]∙H_2_O (B:P = 1:2) (Figure 9b) and Fe[BPO_4_(OH)_2_] (B:P = 1:1) (Figure 9c) are 1D unbranched BPO structures that form helical patterns. The chains coil along an axis and form a tunnel structure, shown by the [∞1(B2P4Φ16)_m_] FBU when viewed along the c-axis (Figure 9b top). 2D layers and 3D frameworks are only observed in a few BPO compositions with B:P = 1:1 (Figure 9d) and 1:2 [130]. In Fe(H_2_O)_2_[B_2_P_2_(OH)_2_]∙H_2_O, borate and phosphate tetrahedra form a six-membered ring [∞2B2P2Φ10] FBU that condense in a 2D layer 6^3^ net-topology (Figure 9d). In these BPOs, the anion units are joined by octahedrally coordinated transition metal polyhedra, MO_6_, forming open framework structures. Gaps or pores formed in the structure create sites that may host alkali ions or free water molecules.

### 5.2. Properties

The open framework structure of BPOs renders them functional for atoms, ions, and molecules’ surface and/or bulk interaction [134]. Hence, BPO finds application in optics, catalysis, separation, ion exchange, microelectronics, and medical diagnosis. Boron phosphate, BPO_4_, has been applied industrially in catalysis applications [135]. These applications necessitate structural and thermal properties characterizations of BPOs (Table 7). Structural properties are typically characterized by XRD. The thermal stability of BPOs varies based on the structure of the BPO but has been reported to remain crystalline at around 200–400 °C. Hydrated BPOs may experience one up to two-step dehydration. Magnetic properties are also being evaluated, especially for optics, catalysis, and separation applications (Table 7). 

### 5.3. Synthesis Routes

BPOs can be synthesized via solid-state, solvothermal, hydrothermal, and microwave-assisted techniques (Figure 9e). Solid-state is a simple yet long-period reaction which yields polycrystalline solids from powdered reactants. Reactants may appear inhomogeneous at the atomic level [139] due to their solid nature. Nucleation and crystal growth of the crystals are facilitated by frequent mixing and high temperatures to supply the necessary thermal energy in the formation reaction [140]. In this method, typical precursors such as boric acid, ammonium dihydrogen phosphate, and alkali carbonates [141,142,143] are mixed in an agate mortar and pestle, placed in a crucible, and preheated to 473–773 K for 9–20 h. The intermediate stage consists of cooling, mixing, and pressing at room temperature in between heat treatments are necessary to break any reactant–product interface and to bring new surfaces into contact. The main reaction step, which typically occurs at 823–1173 K, can take up to weeks. The constant mixture of the reaction system can result in material loss and, consequently, low product yield. Nonetheless, products of solid-state reactions have been reported to be thermally stable until they reach their melting points [127].

BPO synthesized via solvothermal synthesis employs ammonium boron oxide, ammonium dihydrogen phosphate, and potassium tetraborate as precursors in non-aqueous solvents such as glycol and ethanol reacted in a closed vessel at T = 403–493 K for 3–4 days [144,145]. Other high-temperature solvent-based BPO synthesis include flux synthesis [146,147,148] and ionothermal synthesis [149,150]. In flux synthesis, boric acid is supplied in excess, and functions as both precursor and solvent. It participates in the reaction to supply the atoms to build the crystal while facilitating material transport and diffusion in the solution [138]. Precursors such as lithium dihydrogen phosphate, and cupric acetate are mixed with excess boric acid and are reacted at T = 473–513 K for 5–7 days in a stainless-steel vessel. In ionothermal synthesis, ionic liquids (IL) are used as solvents at high temperatures [151]. Due to their exceptional physicochemical properties such as having low viscosity and low vapor pressure, ILs allow high-temperature synthesis with less concerns with volatility. Materials such as boric acid, phosphoric acid, and chlorides are mixed with 1-butyl-1-methylpyrrolidinium bromide to form the IL solvent. The reaction is performed in a closed vessel at T = 383–473 K for 5–6 days.

Hydrothermal synthesis is a solvothermal reaction in an aqueous solution. Unlike the solid-state method that depends on direct contact of the materials at the interface, the dissolution process imparts mobility to the molecules and ions, allowing them to come into contact and react more readily [151]. Precursors of BPO synthesized via hydrothermal method [152,153,154] include phosphoric acid, boric oxide, chlorides, and nitrates. They are dissolved in deionized water in a closed vessel. Homogenous solutions are sealed and heated at T = 433–513 K under autogenous pressures for 5–18 days. After cooling, reaction systems are filtered and washed with hot water to remove any remaining soluble components. The final product is recovered after drying in desiccators at ambient temperature. 

Microwave-assisted synthesis (MAS) addresses the long reaction time of conventional methods. It features low energy consumption and short processing time. It features low energy consumption, short processing time, and low costs. The technique results in unique microstructures and properties [155]. For example, Na_5_[B_2_P_3_O_13_], synthesized via MAS [156,157], has comparable products to those made through hydrothermal synthesis [156]. The hydrothermal route takes 2 days at 423 K while MAS takes 2 min at T < 473 K. However, additional annealing at 373 K for 2 h is required to obtain comparable XRD patterns. Another BPO, (NH_4_)_16_[Zn_16_B_8_P_24_O_96_], synthesized via MAS was heated for 2 h, versus a days-long heating cycle if conducted via solvothermal synthesis. 

## 6. Borosilicates (BSiO)

BSiOs were explored because of the increased interest in tuning the catalytic property of aluminosilicates by replacing Al with other elements such as B, Fe, Ga, and Ge [158]. The first BSiO with a similar isostructure to the NU-1-type zeolitic framework [159] was synthesized by Taramasso et al. [160] in 1980. Its structure and thermal stability were later evaluated by Bellusi et al. [161] in 1990. Synthesis experiments of these modified aluminosilicates concluded that boron was a suitable replacement for aluminum due to its seamless incorporation into the zeolitic structure with the help of templating agents [158]. This exhibits the versatility of BSiO to conform to a wide variety of frameworks. However, the majority of reported BSiOs in the literature are on the discovery of natural BSiO minerals, the applications of synthetic BSiO glasses, and the modification of silicate glasses with boron additives [162,163,164]. However, Li_3_Mo(BO_3_)(SiO_4_), Li_3_V(BO_3_)(SiO_4_), and Li_3_Bi(BO_3_)(SiO_4_) (Table 4) from Li-exchanged Na-based sidorenkite structure were recommended for investigation as cathode materials for secondary LIBs [19]. 

### 6.1. Structures

The isomorphous substitution of aluminum by boron in the parent aluminosilicate zeolite framework led to the analysis of early boron-substituted structures such as reedmergnerite [165], danburite [166], and datolite [167]. The porous 3D framework BSiOs are composed of BO_3_ and/or BO_4_ and SiO_4_ units that are corner linked via oxygen bridges. An increase in the boron content in the well-defined structure decreases the unit cell parameters because of the lower ionic radius of B^3+^ compared to Al^3+^ [168,169]. As BSiOs have similar structures to BPOs, the classification parameters and terms discussed for BPOs in the previous section also apply to BSiOs [108,128,129].

**Figure 10:**
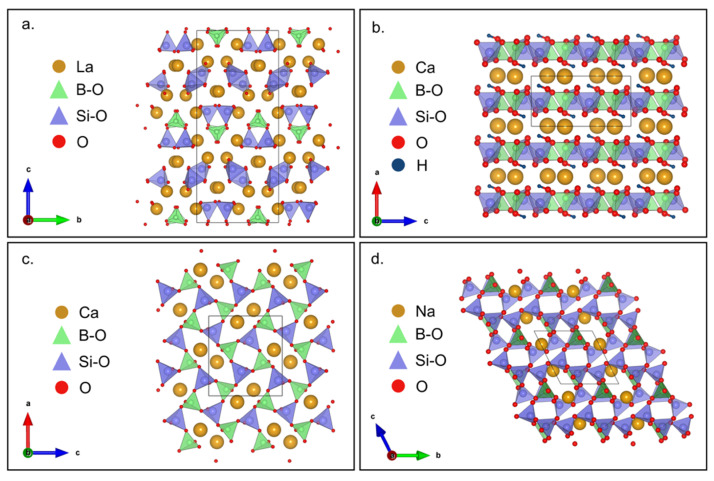
Polyhedral representation of borosilicate structures [41]: (**a**) La_3_BSi_2_O_10_ with isolated units; (**b**) CaBSiO_5_H with layered structure; (**c**) CaB_2_Si_2_O_8_ and (**d**) NaBSi_3_O_8_ with 3D frameworks.

Similar to BPOs, BSiOs have a wide variety of structures with different B:Si ratios (Figure 10). Altering synthetic parameters such as reaction temperature and pressure leads to different polymorphs and arrangements that are classified as isolated, chain, layer, or framework [108]. BSiOs such as La_3_BSiO_10_ (Figure 10a) with isolated groups have their B-O and Si-O bonds in BO_3_ triangles and SiO_4_ tetrahedra, respectively, which, when sharing an oxygen atom, build up the isolated BSiO_6_ group with B-Si-O bonds [170]. Meanwhile, BSiOs like NdBSiO_5_ with chain structures have both the boron and silicon atoms occupy the center space of separate tetrahedra for their bonding with oxygen atoms [171]. Here, the boron, silicon, and oxygen atoms are connected as continuous BSiO_5_ chains with three-membered rings. Furthermore, BSiO such as CaBSiO_5_H (Figure 10b) with layered structure have the typical SiO_4_ tetrahedra but with the uncommon BO_3_(OH) tetrahedra which forms the layered network with four- and eight-membered loops with calcium atoms bonding the layers in between [172].

BSiOs with 3D frameworks offer more combinations of anionic groups. One example is CaB_2_Si_2_O_8_ (Figure 10c), in which silicon and boron bond with oxygen in the usual tetrahedral units but with the terminal tetrahedra being shared with neighboring blocks. This pattern forms corner-sharing Si_2_O_7_ and B_2_O_7_ diortho groups, which build its 3D framework [173]. Another example is NaBSi_3_O_8_ (Figure 10d), in which silicon bonds with oxygen in Si_3_O_8_ double layers that form the Si_3_BO_8_ and five-membered rings in the framework [174]. 

These open-framework compounds with rings and loops [108] offer pathways that are advantageous for the de-/intercalation of cations during charging and discharging. This may result in an increase in power density as the cation can quickly diffuse into porous materials. In addition, silicate structures benefit from the strong Si-O bonds, which can aid in the thermal and mechanical stability of the material during operation. This may result in an increase in the cycle life of the battery as volume expansion and contraction are minimized in the fixed framework.

### 6.2. Properties

Silicon is second to carbon in terms of the number of compounds it can form with other elements, which renders silicates to have diverse structures and properties [128]. While boron is the most suited heteroatom that can be incorporated into the silicate framework, it can only be found in trace amounts in the structure. This makes BSiO characterization difficult in checking the actual level of heteroatom substitution in the tetrahedral sites [158]. Common methods are XRD analysis to check the unit cell parameters as B-O and Si-O bonds have different lengths and NMR techniques to look at the nuclei at different coordination environments (Table 8). Sharp signals on the ^11^B MAS NMR spectrum reflect the presence of symmetric tetrahedral BO_4_ units that can transition to BO_3_ units if heated at elevated temperatures, which is accompanied by structural collapse, as seen in XRD patterns. Similar to zeolites, these BSiOs are known for their porous structures, which are highly desirable for catalytic applications [175,176,177]. The incorporation of boron in the silicate framework lowers its acid strength compared to the parent aluminosilicate. Nonetheless, BSiO can still effectively catalyze reactions that require weak acidity [158,177].

### 6.3. Synthesis Routes

BSiOs have been synthesized hydrothermally in the presence of organic compounds as templating agents in the mixture [168,175,183]. Common silicate precursors are silicon dioxide, tetramethyl orthosilicate, and tetraethyl orthosilicate, while the sole borate precursor is boric acid. Typical synthesis starts with the addition of the silicate precursor in an aqueous solution of the structure-directing agent, such as quinuclidine, ethylenediamine, and pyrrolidine, under an inert atmosphere. This mixture is added to a separate solution containing the borate precursor, which is then stirred to evenly mix the suspension. Afterwards, the combined mixture is placed in an autoclave and heated to 433–493 K for 5–10 days. The product is filtered and washed with water and ethanol. Additional measures such as drying at elevated temperatures of 378–393 K for 4–24 h are also carried out to obtain dry material.

## 7. Borosulfates (BSO)

Seminal works on BSO started as early as 1960s, although nothing much has progressed until recent years. Several BSO of monovalent cations A[B(SO_4_)_2_] (A = Li-Cs, NH_4_, Ag, Tl), and divalent cations M[B(SO_4_)_2_]_2_ (M = Mg-Ba, Pb, Mn, Co-Zn, Cd, Hg) were reported albeit no detailed structural descriptions for these compounds [119]. K_5_[B(SO_4_)_4_], synthesized by Höppe et al. (2012), motivated the discovery of new BSO compounds [184].

K_5_[B(SO_4_)_4_] has a structure analogous to silicates, particularly zunyite, a sorosilicate containing [Si(SiO_4_)_4_]^12−^ ion. Both compounds share the corners of their central tetrahedron T (T = B, Si) with the surrounding tetrahedra X (X = SO_4_, SiO_4_), forming a supertetrahedra, TX_4_ [184]. Due to its structural similarity with silicates and potential properties, studies on new BSO increased in the succeeding years. Netzsch [119] listed a total of only 23 reported BSOs in the literature from 2012 to 2016, and more are expected to be derived as interest in its application increases. Most of the currently known BSO phases have 0D and 1D dimensionality. The analogous structures of BSOs to existing polyanionic cathodes, which have gaps or channels [121] can provide pathways for cation de-/intercalation for secondary battery cathode application.

### 7.1. Structures

**Figure 11:**
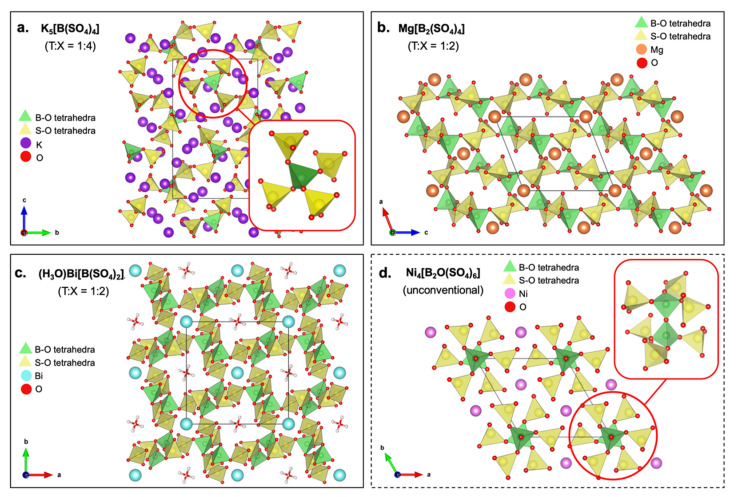
Polyhedral representation of the unit cells of (**a**) K_5_[B(SO_4_)_4_] viewed along [100] direction [184]; (**b**) Mg[B_2_(SO_4_)_4_] viewed along [010] direction [188]; (**c**) (H_3_O)Bi[B(SO_4_)_2_]_4_ [189]; and (**d**) Ni_4_[B_2_O(SO_4_)_6_] viewed along [001] direction [190].

BSO can be classified according to their connection patterns and the dimensionality of their anionic substructure. Based on connection patterns, BSOs can be classical and unconventional. Classical BSOs consist of corner-sharing borate and sulfate tetrahedra which are alternatingly bridged, leading to solely B-O-S bridges. Unconventional BSOs have no perfect alternation of sulfate and borate tetrahedra and may have B-O-B or S-O-S bridges [121]. In terms of dimensionality, Friedrich Liebau’s classification of silicate anions applies to BSO due to their similar structures [128]. Anions of classical BSO can be classified by their B:S ratio. B:S of 1:4 yields 0D (molecular/isolated anions); 1:3 yields 1D (cyclic or infinite chains); 1:2 yields 3D frameworks; and T:X between 1:2 to 1:3 yields 2D layers [121] (Figure 11).

K_5_[B(SO_4_)_4_] has a T:X ratio of 1:4, yielding a 0D anionic substructure where each corner of the borate tetrahedra of [B(SO_4_)_4_]^5−^ anion is surrounded by sulfate tetrahedra (Figure 11a) [119]. Mg[B_2_(SO_4_)_4_] has a T:X ratio of 1:2, yielding a 2D (layer-like) anionic topology (Figure 11b). Such layers are formed by its corner-sharing borate and sulfate tetrahedra arranged alternatingly. Each corner of the borate tetrahedron is connected to adjacent sulfate tetrahedra, while the two corners of sulfate tetrahedra are linked to borate tetrahedra and the other two to terminal O atoms [185]. (H_3_O)Bi[B(SO_4_)_2_]_4_ has a 3D framework due to the B(SO_4_)_4_ supertetrahedra bridged by four surrounding sulfate tetrahedra (Figure 11c). This network forms Vierer ring channels which host the bismuth cations and the Achter ring channels which contain the oxonium cations [186]. Ni_4_[B_2_O(SO_4_)_6_], an example of unconventional BSO, has three corners of borate tetrahedron shared with three sulfate tetrahedra, and the remaining corner is shared with another borate tetrahedron (Figure 11d). Due to the arbitrary dimensionality of unconventional BSO, Ni_4_[B_2_O(SO_4_)_6_] may exhibit a 1D or 3D structure depending on the degree of condensation [187]. For all these BSO classifications, the common structural theme is that they are composed of infinite layers or networks of borate and sulfate tetrahedra with channels or spaces where the metal ion or cation can insert. These structures show similarities with current good polyanionic cathodes, which suggests BSO’s great potential in secondary battery applications.

### 7.2. Properties

Like BPOs, BSOs are classified as silicate-analogous materials due to their corner-sharing borate and sulfate tetrahedra TX_4_ (T = tetrahedral center; X = corners) [185]. The lack of an inversion center in its tetrahedra [186] and its ability to host optically active ions [185] gives this compound excellent optical properties (Table 9). Moreover, the possible presence of BO_3_ moiety can act as a Lewis acid center which is a significant property in catalysts [119]. These applications necessitate the characterization of synthesized BSO which include XRD, thermal analysis, and optical spectroscopy (Table 9). 

### 7.3. Synthesis Routes

There are two main routes for the synthesis of BSOs – solid-state reactions and solvent-driven reactions. In solid-state synthesis, metal disulfates (e.g., K_2_S_2_O_7_) or hydrogensulfate hydrates (e.g., NaHSO_4_·H_2_O) are used as a source of sulfate fragments. These salts are ground together with suitable boron sources such as boron oxide B_2_O_3_, metaboric acid HBO_2_, or boric acid H_3_BO_3_. Such reactions at elevated temperatures (400 °C) lead to the formation of alkali borosulfates [119].

Solvent-driven reactions can be further differentiated into precipitation and solvothermal synthesis. Almost 48% of the known BSO phases today are synthesized using precipitation reactions which can occur under closed or open systems. Such reactions occur by dissolving metal sulfate and boric acid in concentrated sulfuric acid, followed by heating. Meanwhile, solvothermal synthesis accounts for a minor fraction (13%) of the BSO phases in the literature [119]. These BSOs are synthesized from the superacid H_5_[B(SO_4_)_4_], boron hydroxide, or boron oxide and their corresponding metal powder, metal oxide, or metal carbonate. Oleum was added to these, and the mixtures were subsequently placed in a sealed silica glass ampule and heated to relatively high temperatures [120]. Among the three methods, solvothermal synthesis is the most promising as it promotes condensation reaction while avoiding undesirable reactions brought by sensitivity towards moisture [121].

## 8. Outlook and Target Structures

In the development of advanced cathode materials, the composition of the compound is also an important factor. For the transition metal component, aside from the redox reactivity, the global supply and cost are considerations to ensure that the chemistry is viable in the long term. In designing cathodes, it is preferable to select abundant and low-cost elements, in contrast to present components such as Co. Thus, highlighted in the previous sections were borate-based compounds composed of earth-abundant transition metals such as Fe and Ni.

To identify potential novel borate-based cathode materials, a computational and experimental complementary approach may be taken. Based on screening criteria and using DFT calculations similar to what is outlined by Ceder et al. [19], stable compounds meeting minimum performance (e.g., >150 mAh g^−1^ theoretical specific capacity in LIB) and composition (use of earth-abundant metals, e.g., Fe or Ni) requirements can be identified from a large pool of chemistries. Chen et al. [92] predicted the stability of carbonophosphates from over 10,000 database and data-mined compounds via high-throughput computing, which they validated via experimental synthesis and characterization. Conversely, ab initio techniques may also be used as a tool to corroborate and elaborate experimental observations, such as the composition and structure, and measurements from x-ray absorption spectroscopy, electron energy-loss spectroscopy, nuclear resonance spectroscopy, and transmission electron spectroscopy [191]. 

Borate-based compounds with transition metal-borate-based chemistries other than Fe and Ni in the literature should be explored. These other chemistries serve as model phases whose transition metal can be substituted with earth-abundant metals, such as Fe and Ni. Model compounds of BPO [112,126,192,193,194,195,196,197,198,199,200,201] (Table S1), BSiO [181,202,203] (Table S2), and BSO [185,186,187] (Table S3) with transition metal (M = Co, Mn, Cu, and Zn) compositions are found in the literature. These model phases feature 2D layered and 3D open framework channel-forming structures that can host alkali metal ions either in the interlayers or in the pores or pockets of the structure. The suggested synthesis routes to arrive at these isostructural target compounds are mostly hydrothermal techniques. 

The limited literature on borate-based mixed polyanion cathode materials showed varying results and limited cycling depending on the intercalating ion and electrolyte [109,110]. Battery systems with borate-based cathodes were expected to have low energy densities due to the large building units of borate, phosphate, silicate, and sulfate polyhedral. Nevertheless, the limited literature and identified limitations open opportunities for further exploration in cathode modification, such as surface coating, particle size reduction, doping, and defect engineering [21,204,205].

Aside from the transition metal, the intercalating species is also an important factor. The discussion on the suggested borate-based mixed polyanion cathode materials focuses on their application for monovalent-ion batteries. A strategy to increase capacity is to utilize multivalent ions such as Zn^2+^, Mg^2+^, Ca^2+^, etc., which have been intercalated in metal oxides consisting of multivalent transition metals such as V^x+^ (x = 2−5) [206,207] and Mn^x+^ (x = 2−7) [208], chalcogenides with chevrel [209] and layered structures [210,211], polyanions [212,213], and metal organic frameworks [214]. However, the literature on the use of mixed polyanion cathodes, including borate-based compounds for multivalent-ion batteries, is limited [215,216]. While multivalent-ion batteries have a similar operating mechanism to monovalent-ion batteries, the high electronegativity, charge density, and valence electron potential of multivalent ions result in sluggish mobility and a high energy barrier during ion deintercalation [216,217,218]. These kinetic limitations hinder the smooth adaption of host frameworks of mature and widely researched monovalent-ion batteries for multivalent-ion batteries. However, given the variety of stable structures with wide channels of borate-based materials, further theoretical and experimental validation might still be worth exploring to investigate the intercalation mechanism of multivalent ions in mixed polyanion cathode materials.

## 9. Conclusions

Aside from enabling the transition towards renewable energy, energy storage systems, such as rechargeable LIBs and sodium-ion batteries (SIBs), improve the standard of living through their many innovative and practical applications. These rechargeable battery technologies enable renewable energy and the electrification of vehicles. However, as the use of batteries increases, the demand for improved performance and lower cost also increases. While improving currently available battery materials is one way to address this problem, another approach is to develop new materials for battery components, such as the cathode, to bring new solutions into the mix.

This review has proposed the families of borate-based compounds as potential cathode materials by outlining their history, structures, properties, performance, and synthetic routes. These compounds have compositions that can take advantage of the redox reactivity and low cost of earth-abundant metals in their chemistries. They exhibit a wide variety of structures and properties that are regarded for optical and catalytic applications. However, there is a lack of research and information on their electrochemical properties. Thus, this review sought to develop the motivation to explore these compounds for cathode applications. As their crystal structures are like those of commercial cathode materials in terms of the presence of alkali metal ion host sites and ion diffusion pathways, these proposed compounds are predicted to have highly desirable properties for battery applications. Furthermore, mixed polyanion compounds are predicted to have high and tunable potentials, making them good cathode materials. Thus, the families of borate-based compounds open avenues for the discovery and development of advanced cathode materials. 

## Figures and Tables

**Table 1:** Range of reversibility and theoretical specific capacity and capacity density of early dichalcogenide-based cathodes (Data retrieved from Ref. [33]. Copyright 1994, Elsevier).

Cathode Material	Reversible Range (Δx)	Specific Capacity (Ah kg^−1^)	Capacity Density (Ah I^−1^)
Li*_x_*TiS_2_	1.0	239	782
Li*_x_*MoS_2_	0.8	134	678
Li*_x_*NbSe_3_	3.0	244	2121

**Table 2:** Summary of the advantages and disadvantages of different oxide cathodes.

Type of Oxide Cathode	Electronic Conductivity ^1^	Structure	Stability	Sustainability ^1^	Ref.
Layered	(+)	close-packed; high density	acid leaching of transition metals	(−)	[59,60]
Spinel	(+)	close-packed; high density		(−)	[59,60]
Polyanion	(−)	low density	better safety due to tightly bound O to P, S, or Si	(+)	[5]

^1^ A (+) symbol indicates relatively good performance while (−) indicates poor performance.

**Table 3:** Summary of electrochemical performances of different oxide cathodes.

Crystal Structure	Compound	Cell Voltage (V)	Specific Capacity (mAh g^−1^) Theoretical/Actual	Remarks	Ref.
Layered	LiCoO_2_	3.8	274/137	Excellent cycling performance	[73]
				High working voltage	
	LiNiO_2_	3.7	275/160	High specific capacity	[74]
				Low thermal stability	[75]
	LiMnO_2_	3.3	285/130	Low structural stability	[76]
				Favorable transformation to spinel structure	[77]
	LiNi_0.65_Co_0.15_Mn_0.2_O_2_	4.3	186.5	High working voltage and capacity Poor cycling efficiency, rate performance, and thermal stability	[52]
	LiTiS_2_	1.9	239/235	Significant capacity fade	[78]
Spinel	LiMn_2_O_4_	4	145/120	Significant capacity fade	[79]
				Poor cycling stability	[80]
	LiTi_2_O_4_	1.5	240	Low operating voltage	[81]
				Good superconductivity	[82]
	LiV_2_O_4_	1.2	155/100	Low structural stability	[83]
				Significant capacity fade at different voltages	
	LiCo_2_O_4_	3.9	142	Low structural stability	[84]
				Favorable transformation to layered structure	
Olivine/ polyanion	LiFePO_4_	3.5	170/ 165	Good cycling stability	[69]
				High rate capability	[85]
				Excellent thermal stability	
	LiCoPO_4_	4.8	167	Poor cycling stability	[86]
				Low coulombic efficiency	
				Poor ionic conductivity	
	LiMnPO_4_	4.1	171/140	High capacity fade at different discharge rates	[87]
	LiFeSO_4_F	3.9	151/140	High rate capability	[88]
	Li_2_FeSiO_4_	2.8	166/140	Low operating voltage	[89]
				Good electrochemical process reversibility	
	LiMnP_2_O_7_	4.0	120	High mechanical stability	[90]
	Li_2_CoPO_4_F	5.0	310	High electrolyte decomposition	[91]
	Li_3_FeCO_3_PO_4_	3.3	115/110	Capacity increase after ball milling with carbon	[92]

**Table 4:** Computed LIB properties for the Na-compounds of carbonophosphates [(CO_3_)(PO_4_)] and carbonosilicates [(CO_3_)(SiO_4_)] stable in sidorenkite structure (viz., likely to be synthesizable via Li-Na exchange) as well as that of borate-based compounds close to stability in Na sidorenkite structure (<30 meV at^−1^) in comparison to two commercial cathode materials (Data retrieved from [19]. Copyright 2011, The Royal Society of Chemistry).

Compound	Formula	Voltage (V)	Capacity (mAh g^−1^)	Specific Energy (Wh kg^−1^)	Energy Density (Wh I^−1^)	Change in Volume (% per *e^−^*)
(CO_3_)(PO_4_)	Li_3_Mn(CO_3_)(PO_4_)	3.3; 4.1	232	859	2375	1.20
	Li_2_V(CO_3_)(PO_4_)	3.5; 4.4	243	969	2604	0.90
(CO_3_)(SiO_4_)	Li_3_V(CO_3_)(SiO_4_)	3.0; 3.7	239	799	2183	0.32
	Li_3_Mo(CO_3_)(SiO_4_)	2.6; 3.5; 3.5	299	966	2989	0.24
BO_3_-based	Li_3_Mn(BO_3_)(PO_4_)	4.1	117	473	1315	3.50
	Li_3_Mo(BO_3_)(PO_4_)	2.9; 3.7; 3.7	296	1024	3200	0.21
	Li_3_Cr(BO_3_)(PO_4_)	4.2; 5.1; 5.1	354	1705	4814	0.59
	Li_3_V(BO_3_)(PO_4_)	3.4; 4.3	237	904	2487	0.44
	Li_3_Mo(BO_3_)(SiO_4_)	3.2; 3.7	200	692	2167	0.62
	Li_3_Fe(BO_3_)(PO_4_)	4	116	468	1330	0.61
	Li_3_Bi(BO_3_)(PO_4_)	4.3; 4.6	140	624	2444	0.01
	Li_3_V(BO_3_)(SiO_4_)	3.6	120	428	1172	1.30
	Li_3_Bi(BO_3_)(SiO_4_)	3.6	70	286	1039	2.60
Control	LiFePO_4_	3.4	170	544	1959	6.80
	LiCoO_2_	4	155	620	3100	1.80

**Table 5:** Electrochemical performance of the BPO cathode materials.

Cathode Material	Space Group	Intercalating Ion	Cathode Potential (V)	Specific Capacity (mAh g^−1^)	C-Rate	Remarks	Ref.
LiFe-BPO	*P*6_1_22	Li^+^	3.06	67.5	C/50	Higher reversibility compared to LiFe-BPO versus. Na^+^/Na. Achieved ~80% of theoretical capacity.	[109]
LiFe-BPO	*P*6_1_22	Na^+^	2.76	66.5	C/50	Achieved ~82% of its theoretical capacity. Observed a capacity loss of 9% after the third cycle.	[109]
NaFe-BPO	*P*6_1_22	Na^+^	2.9	66	C/20	Became almost electrochemically inactive by its 10th cycle.	[110]
NHFe-BPO	*P*6_5_22	Na^+^	2.9	80		Capacity drops 60% after the 40th cycle.	[110]

**Table 6:** Electrochemical performance of the BO_3_^3−^ doped LiFeSiO_4_ cathode materials.

Cathode	Space Group	Initial Capacity (mAh g^−1^)	Cathode Potential (V)	Charge Transfer Impedance R_ct_ (Ω)	Li Diffusion Coefficient D_Li_^+^ (cm^2^ s^−1^)	Ref.
Li_2_Fe_0.98_Mg_0.02_(SiO_4_)_0.97_(BO_3_)_0.03_/C	*P*2_1_/*n*	138	~3	3546.0	2.68 × 10^−15^	[117]
Li_2_Fe_0.98_Ag_0.02_(SiO_4_)_0.99_(BO_3_)_0.01_/C	*P*2_1_/*n*	150.8	~3	933.6	3.09 × 10^−16^	[118]

**Table 7:** Properties of different BPO known phases featuring earth-abundant metals.

Compound	X-ray Diffraction	Thermal Analyses	Magnetic Susceptibility	Infrared Spectroscopy	Application	Ref.
*M* [BPO_4_(OH)_2_] (*M* = Mn, Fe, Co)	Chiral space group *P*3_2_21 or *P*3_1_21 Helical *M*O_6_-chains along [001] Vierer BPO single chains perpendicular to [001]	One-step dehydration thermally stable at least up to ~458.85 to 492 °C	µ_eff_ values typical for pure (high-spin) *M*^II^ compounds at lower temperatures, ø(T) curves indicate low-dimensional antiferromagnetic correlations		Nonlinear optics	[136]
*M*^I^*M*^II^(H_2_O)_2_[BP_2_O_8_] ∙H_2_O (*M*^I^ = Na, K; *M*^II^ = Mg, Mn, Fe, Co, Ni, Zn)	Space group *P*6 hexagonal PO_4_ and BO_4_ tetrahedral helical ribbons through common vertices	Two-step dehydration thermal stability varies between 180 °C to 305 °C			Catalysis, separation	[137]
Fe(H_2_O)_2_BP_2_O_8_∙H_2_O	Space group *P*6_5_22 zeolite-type, tetrahedral, chiral framework topology	First dehydration step at 100–235 °C and second at 500 °C Structure is crystalline at 235 °C and amorphous at 400 °C Unit cell volume decreases during heating	Paramagnetic down to 5 K of the Curie-Weiss type antiferromagnetic interactions between iron centers		Catalysis, separation, ion exchange	[131]
*M*(H_2_O)_2_[B_2_P_2_O_8_ (OH)_2_]∙H_2_O (*M* = Fe, Co, Ni)	n space group *P*2_1_/*c* wavy 6_3_ net 2D arrangement of distorted corner sharing PO_4_ and HBO_4_	Mass loss is between ~97 to 327 °C. Framework starts to decompose at ~247 °C	Magnetic behavior below 40 K (zero-field splitting and/or high-spin/low-spin transition)		Sorption, separation, catalysis, optics	[133]
*M*^III^_2_BP_3_O_12_ (*M* = Fe, In)	Space group *P*6_3_/*m* hexagonal 3D architectures of corner-sharing M_2_O_9_ and B(PO_4_)_3_ units		Strong antiferromagnetic coupling dominates the exchange between iron atoms	Transparent range of 4000–1700 cm^−1^ optical band gaps of 5.39 eV (In_2_BP_3_O_12_) and 3.52 eV (Fe_2_BP_3_O_12_)	Sorption, separation, catalysis, ion exchange, optics	[138]

**Table 8:** Properties of different BSiO structure types.

Compound	X-ray Diffraction	Thermal Analysis	Nuclear Magnetic Resonance	Application	Ref.
LaBSiO_5_	Space group *P*3_1_ Six-membered rings composed of BO_4_ and SiO_4_ tetrahedra			Ultraviolet nonlinear optical applications	[178]
Sr_3_B_2_Si_2_O_8_	Space group *Pnma* Chain of SiO_4_, BO_4_, and BO_3_ polyhedra	Decomposition at 1043 K Maximal thermal expansion along [010]		Fabrication of glaze glass coatings	[179]
CaBSiO_4_(OH)	Space group *P*2_1_/*c* Two sets of alternating layers; first layer of 8- and 4-membered rings; second layer of Ca polyhedral in 6-membered ring	Axial expansion along [100] and [[010]] Volume thermal expansion coefficient of 1.5 × 10^−5^ K^−1^		Geochemical marker	[180]
NaCa_5_(BO_3_)(SiO_4_)_2_	Space group *P*2_1_/*c* Framework of isolated BO_3_ and SiO_4_ polyhedra connected by NaO_7_ polyhedra	No significant weight loss until 1513 K		Birefringent and nonlinear optical applications	[181]
SrB_2_Si_2_O_8_	Space group *Pnma* No phase transformation until 1173 K	Expands isotropically Volume thermal expansion coefficient of 25.4 × 10^−6^ °C^−1^		Phosphors	[182]
K_16_[B_16_Si_32_O_96_]	Space group $Ia\bar{3}d$ ANA structure type Isostructural with leucite	Weight loss at 443–504 K due to hydrated phase and adsorbed water High thermal stability compared to other zeolite-type borosilicates due to its potassium ions	Sharp signal on the ^11^B spectrum showed symmetric tetrahedral (BO_4_) units Calcination did not change ^11^B spectrum Broad signal on ^29^Si spectrum due to short T_2_ of the nuclei		[175]
Na_5_B_5_Si_49_O_108_	Space group $R\bar{3}m$, hexagonal Levyne structure type Small structural collapse after calcination at 823 K	Three-step weight loss at 541 K, 775 K, and 845 K due to elimination of organic material in the framework	Symmetric tetrahedral unit BO_4_ from sharp signal Calcination at 823 K led to transition from tetrahedral to trigonal symmetry		[176]

**Table 9:** Properties of different BSO known phases featuring earth-abundant metals.

Compounds	X-ray Diffraction	Thermal Analysis	Optical Spectroscopy	Infrared Spectroscopy	Applications	Ref.
M[B_2_(SO_4_)_4_] M = Mg, Co	Space group *C*2/*c* Infinite anionic layers parallel to (100) plane	Start to decompose above 450 °C	Reflectance spectra similar and typical of Co^2+^ ions in an almost undistorted oxoanionic coordination.	1195 cm^−1^: asymmetric stretching (S-O _terminal_) 1170–1014 cm^−1^: asymmetric and symmetric stretching (B-O) 696–553 cm^−1^: asymmetric bending (O-S-O, O-B-O, S-O-B)	Used in optical materials	[185]
(H_3_O)Bi[B(SO_4_)_2_]_4_	Space group $I\bar{4}$ 3D network consists of SO_4_ supertetrahedra sharing all four corners with other SO_4_	Stable up to 180 °C before decomposition	UV region absorption edge	1400–400 cm^−1^: borate and sulfate tetrahedra presence 840–1040 cm^−1^: B-O stretching >1100 cm^−1^: S-O stretching	Used in luminescent materials	[186]
*M*_4_[B_2_O(SO_4_)_6_] *M* = Mg, Mn, Co, Ni, Zn	Space group $P\bar{3}$ Layers of edge-sharing [B_2_O(SO_4_)_6_]^8−^ anions and MO_6_ octahedra	Starts to decompose around 500 °C	High reflectance around 620–420 nm; pink body color of the compound	1150–1420 cm^−1^: asymmetric SO_4_ stretching 980–1080 cm^−1^: asymmetric BO_4_ stretching 750–820 cm^−1^: S-O-B and B-O-B stretching 430–660 cm^−1^: BO_4_ and SO_4_ bending	Used as host material for phosphors	[187]

## Data Availability

Not applicable.

## Supplementary Materials

The following supporting information can be downloaded at: https://www.mdpi.com/article/10.3390/molecules27228047/s1, Table S1: Model metal-BPO compounds and the suggested synthetic routes to obtain earth-abundant metal target isostructures; Table S2: Model metal-BSiO compounds and the suggested synthetic routes to obtain earth-abundant metal target isostructures; Table S3: Model metal-BSO compounds and the suggested synthetic routes to obtain earth-abundant metal target isostructures.
