# Supplementary material for: Borate-Based Compounds as Mixed Polyanion Cathode Materials for Advanced Batteries
Source: Molecules. 2022 Nov 19;27(22):8047. doi: 10.3390/molecules27228047 (PMC9695605; doi:10.3390/molecules27228047)
Supplement: Supplementary file 1 [file molecules-27-08047-s001.zip › molecules-2014787-supplementary.pdf]

Supporting Information

Borate-based Compounds as Mixed Polyanion Cathode Materials for Advanced Batteries

Giancarlo Dominador D. Sanglay [1,2,3], Jayson S. Garcia [1,2,3], Mecaelah S. Palaganas [1,3], Maurice Sorolla II [3,4], Sean See [3,5], Lawrence A. Limjuco [1,3,6] and Joey D. Ocon [1,2,3,*]

[1] *Laboratory of Electrochemical Engineering (LEE), Department of Chemical Engineering, University of the Philippines Diliman, Quezon City 1101, Philippines*

[2] *Energy Engineering Program, National Graduate School of Engineering, College of Engineering, University of the Philippines Diliman, Quezon City 1101, Philippines*

[3] *DOST-NICER Advanced Batteries Center, University of the Philippines Diliman, Quezon City 1101, Philippines*

[4] *Department of Chemical Engineering, University of the Philippines Diliman, Quezon City, 1101, Philippines*

[5] *Institute of Chemistry, University of the Philippines Diliman, Quezon City 1101, Philippines*

[6] *College of Engineering, University of Southeastern Philippines, Obrero, Davao City 8000, Philippines*

Corresponding author: jdocon@up.edu.ph

Table S1. Model metal-BPO compounds and the suggested synthetic routes to obtain earth-abundant metal target isostructures.

Model Structures	Structure Description	Target Isostructure	Suggested Synthetic Routes	Reference
$M[BPO_4(OH)_2]$<br>M = Mn, Fe, Co	A 3D structure formed by edge-sharing helical MO_6 chains wound around 3_1 or 3_2 screw axes interconnected by BPO single chains of alternating $BO_2(OH)_2$ and PO_4 tetrahedra	$Ni[BPO_4(OH)_2]$	Hydrothermal method:<br><br>$NiCl_2{\cdot}4H_2O + H_3BO_3$ + DABCO + H_3PO_4<br>$\xrightarrow{170\ ℃\ H_2O}$<br>$Ni[BPO_4(OH)_2]$<br><br>DABCO = 1,4-diazobicyclo[2.2.2] octane	[1]
$Fe_2BP_3O_{12}$	A 3D network formed by BO_3 triangular planar structures corner sharing three PO_4 tetrahedra to form a zero-dimensional (0D) anionic unit of $[BP_3O_{12}]^{6-}$. The BPO units are further connected by the FeO_6.	$Ni_2BP_3O_{12}$	Flux method<br><br>Cs_2CO_3 (flux) + $H_3BO_3 + NH_4H_2PO_4$ + NiO<br>$\xrightarrow{850\ ℃\ H_2O}$<br>$Ni_2BP_3O_{12}$	[2]
$(Co_{0.6}Mn_{0.6})_2(H_2O)[BP_3O_9(OH)]$<br>$Mg_2(H_2O)[BP_3O_9(OH)_4]$	Isostructural phases constructed from infinite chains of alternately connected distorted $M^{II}O_4(OH)(H_2O)$ and $M^{II}O_5(OH)$ octahedra. The M^{II} chains are interconnected by BPO tetrahedra, resulting in a 3D framework structure.	$M_2(H_2O)[BP_3O_9(OH)_4]$	Hydrothermal method:<br><br>$M(OH)_2 + H_3BO_3 + H_3PO_4$<br>$\xrightarrow{190\ ℃\ H_2O}$<br>$M_2(H_2O)[BP_3O_9(OH)_4]$	[3]<br><br>[4]
$Co_5BP_3O_{14}$	The structure is composed of triangular planar BO_3 corner sharing PO_4 tetrahedra forming $BPO_6{}^{4-}$ dimers. Layers of the dimers are separated by double layers of isolated PO_4 tetrahedra. There are five Co sites in the structure joining the BPO layers together	$M_5BP_3O_{14}$<br>M = Fe, Ni	High-Temperature Flux Method:<br><br>$MCO_3{\cdot}xH_2O + B_2O_3$(flux) + H_3PO_4<br>$\xrightarrow{950\ ℃}$<br>$M_5BP_3O_{14}$	[5]
$Cu[B_2P_3O_{12}(OH)_3]$	The 3D framework is composed of a BPO chain formed by corner-sharing borate and phosphate tetrahedra in 4-membered tetrahedral rings and a Cu zigzag chain in alternating trigonal bipyramid and octahedral coordination. The chains are alternately stacked forming a parallel rod stacking matrix. Additional PO_4 tetrahedron are found in between the copper polyhedral chains further joining the structure.	$M[B_2P_3O_{12}(OH)_3]$<br>M = Fe, Ni	Hydrothermal method:<br><br>$MCl_2{\cdot}xH_2O + H_3BO_3 + NH_4H_2PO_4$<br>$\xrightarrow{170\ ℃\ H_2O}$<br>$M[B_2P_3O_{12}(OH)_3]$	[6]

$Zn[BPO_4(OH)_2]$	An open framework structure built from unbranched vierer-single BPO chains. The chains are interconnected by Zn tetrahedra to form a 3D structure	$M[BPO_4(OH)_2]$ M = Fe, Ni	Hydrothermal method: $MO + B_2O_3 + P_2O_5 \xrightarrow{170\ ^\circ C\ H_2O} M[BPO_4(OH)_2]$	[7]
$LiCu_2BP_2O_8(OH)_2$	An open framework structure built up of layers formed by BO_4 and PO_4 tetrahedra joined together by CuO_6 and LiO_6. The Li lies in a center of symmetry.	$LiM_2BP_2O_8(OH)_2$ M = Fe, Ni	Hydrothermal method: $M(NO_3)_2{\cdot}xH_2O + Li_2B_4O_7 + H_3PO_4 \xrightarrow{170\ ^\circ C\ H_2O} LiM_2BP_2O_8(OH)$	[8]
$Na_5(H_3O)\{M_3[B_3O_3(OH)]_3(PO_4)_6\}{\cdot}2H_2O$ M = Mn, Co, Ni	An open framework structure built up of layers formed by BO_4 and PO_4 tetrahedra joined together by CuO_6 and LiO_6. The Li lies in a center of symmetry.	$Na_5(H_3O)\{Fe_3[B_3O_3(OH)]_3(PO_4)_6\}{\cdot}2H_2O$	Hydrothermal method: $FeCl_2{\cdot}xH_2O + H_3BO_3 + Na_2HPO_4{\cdot}12H_2O \xrightarrow{200\ ^\circ C\ H_2O} Na_5(H_3O)\{Fe_3[B_3O_3(OH)]_3(PO_4)_6\}{\cdot}2H_2O$	[9]
$NaFe[BP_2O_7(OH)_3$	A 3D structure is formed by metal phosphate Kagome lattice layers are joined together by triborates. The 3D framework forms channel structures joined by MO_6. Na+ reside in pockets in the structure.	$NaNi[BP_2O_7(OH)_3]$	Hydrothermal method: $NiCl_3{\cdot}xH_2O + Na_2B_4O_7{\cdot}10H_2O + Na_2HPO_4{\cdot}2H_2O \xrightarrow{170\ ^\circ C\ H_2O} NaNi[BP_2O_7(OH)_3]$	[10]
$CaCo(H_2O)[BP_2O_8(OH)]{\cdot}H_2O$	Structure is built on isolated BPO anions joined by Fe octahedrons. Na^+ is 8-fold coordinated to O forming cubes.	$MgM(H_2O)[BP_2O_8(OH)]{\cdot}H_2O$ M = Fe, Ni	Hydrothermal method: $Mg(OH)_2 + M(C_2H_3O_2)_,{\cdot}xH_2O + B_2O_3 + H_3PO_4 \xrightarrow{170\ ^\circ C\ H_2O} MgM(H_2O)[BP_2O_8(OH)]{\cdot}H_2O$	[11]
$CaFe[BP_2O_7(OH)_3]$	Layers built of BPO oligomers, interconnected by Co octahedra. Inter-layer connections are by Ca octahedra.	$MgFe[BP_2O_7(OH)_3]$	Hydrothermal method: $Mg(OH)_2 + FeCl_2{\cdot}xH_2O + B_2O_3 + H_3PO_4 \xrightarrow{170\ ^\circ C\ H2O} MgFe[BP_2O_7(OH)_3]$	[12]

Table S2. Model metal-BSiO compounds and the suggested synthetic routes to obtain earth-abundant metal target isostructures.

Model Structures	Structure Description	Target Isostructure	Suggested Synthetic Routes	Reference
$NaCa_5BO_3(SiO_4)_2$	A tangled 3D network of isolated BO_3 triangles and SiO_4 tetrahedra. Calcium bonds with 7-9 oxygen atoms which build up the 3D Ca-O framework	$NaM_5BO_3(SiO_4)_2$<br>M = Fe, Ni	Solid-state method<br><br>$Na_2CO_3 + MCO_3 + B_2O_3 + SiO_2 \xrightarrow{890\ ℃} NaM_5BO_3(SiO_4)_2$	[13]
$CaBSiO_5H$	Layers of B-Si-O networks connected by Ca atoms. SiO_4 and $BO_3(OH)$ tetrahedra form the $BSiO_4(OH)$ apophylite layers with four- and eight- membered loops	$MBSiO_4(OH)$<br>M = Fe, Ni	Hydrothermal method<br><br>$MO + B_2O_3 + SiO_2 \xrightarrow{250\ ℃\ H_2O} MBSiO_4(OH)$	[14]
$BaB_2Si_2O_8$	A 3D structure of Si_2O_7 and B_2O_7 diortho groups that form the $Si_2B_2O_8$ anorthite-like framework	$MB_2Si_2O_8$<br>M = Fe, Ni	Solid-state method<br><br>$MCO_3 + H_3BO_3 + SiO_2 \xrightarrow{850\ ℃} MB_2Si_2O_8$	[15]

Table S3. Model metal-BSO compounds and the suggested synthetic routes to obtain earth-abundant metal target isostructures.

Model Structures	Structure Description	Target Isostructure	Suggested Synthetic Routes	Reference
$M[B_2(SO_4)_4]$<br>M = Mg, Co	α-$M[B_2(SO_4)_4]$ - Comprises of infinite anionic layers consisting of alternating, corner sharing borate and sulfate tetrahedra. Each borate tetrahedron is bonded to adjacent sulfate tetrahedra while the sulfate tetrahedra have two borate bridging oxygen atoms and two terminal oxygen atoms. The BSO layers are joined together by octahedral binding M.<br>β-$M[B_2(SO_4)_4]$ - Consists of alternating corner-sharing borate and sulfate tetrahedra that form a layer of adjacent Vierer and Zwölfer rings. M ions are found on the corners and at the centre of the unit cell bound octahedrally within the Zwölfer ring and with two oxygen atoms from adjacent layers.	$M[B_2(SO_4)_4]$<br>M = Fe, Ni	Solvothermal method:<br>$M(CO_3)_3{\cdot}xH_2O + B_2O_3 + H_2SO_4 \xrightarrow{180\ ℃} M[B_2(SO_4)_4]$	[16]
$(H_3O)Bi[B(SO_4)_2]_4$	Consists of all-corner sharing borate and sulfate supertetrahedra forming a 3D network. Vierer and Achter rings are found in the structure forming channels. The Vierer ring channels host the bismuth, while the Achter ring channels the H_3O^+.	$NaM[B(SO_4)_2]_4$<br>M = Fe, Ni	Solvothermal method:<br>$NaCl + MCl_3{\cdot}xH_2O + H_3BO_3 + H_2SO_4 \xrightarrow{200\ ℃} NaM[B(SO_4)_2]_4$	[17]
$M_4[B_2O(SO_4)_6]$<br>M = Mg, Mn, Co, Ni, Zn	α-$M_4[B_2O(SO_4)_6]$ - Comprises of layers of open-branched quadruple tetrahedra edge-sharing $[B_2O(SO_4)_6]^{8-}$ anions. The BSO layers are joined by the octahedra forming M_2O_9 face-sharing dimers forming a 3D network.<br><br>β-$M_4[B_2O(SO_4)_6]$ - Comprises the same fundamental building units as the α-polymorph; however, the BSO moieties alternate between staggered and eclipsed conformations	$Fe_4[B_2O(SO_4)_6]$	Solvothermal method:<br>$H[B(HSO_4)_4] + FeO \xrightarrow{200\ ℃} Fe_4[B_2O(SO_4)_6]$	[18]

References

1. Boy, I.; Schäfer, G.; Kniep, R. Crystal Structure of Sodium Iron(II) Diaqua Catena-[Monoboro-Diphosphate] Monohydrate, $NaFe(H_2O)_2[BP_2O_8]\cdot H_2O$, and of Potassium Iron(II) Diaqua Catena-[Monoboro-Diphosphate] Hemihydrate, $KFe(H_2O)_2[BP_2O_8]\cdot 0.5H_2O$. *Z. Kristallogr. - New Cryst. Struct.* **2001**, *216*, 13–14, doi:10.1524/ncrs.2001.216.14.13.
2. Ozdil, Y. Synthesis and Characterization of Rare-Earth Borophosphates. M.S. Thesis, The Middle East Technical University, Turkey, 2004.
3. Huang, Y.-X.; Prots, Y.; Kniep, R. Crystal Structure of Cobalt Manganese Monoaqua Catena-[Monohydrogenborate- Tris(Hydrogenphosphate)], $(Co_{0.6}Mn_{0.4})_2(H_2O)[BP_3O_9(OH)_4]$. *Z. Kristallogr. - New Cryst. Struct.* **2009**, *224*, 371–372, doi:10.1524/ncrs.2009.0162.
4. Ewald, B.; Öztan, Y.; Prots, Y.; Kniep, R. Structural Patterns and Dimensionality in Magnesium Borophosphates: The Crystal Structures of $Mg_2(H_2O)[BP_3O_9(OH)_4]$ and $Mg(H_2O)_2[B_2P_2O_8(OH)_2]\cdot H_2O$. *Z. Anorg. Allg. Chem.* **2005**, *631*, 1615–1621, doi:10.1002/ZAAC.200500061.
5. Bontchev, R.P.; Sevov, S.C. $Co_5BP_3O_{14}$: The First Borophosphate with Planar BO_3 Groups Connected to PO_4 Tetrahedra. *Inorg. Chem.* **1996**, *35*, 6910–6911, doi:10.1021/ic960808d.
6. Duan, R.; Liu, W.; Cao, L.; Su, G.; Xu, H.; Zhao, C. A New Copper Borophosphate with Novel Polymeric Chains and Its Structural Correlation with Raw Materials in Molten Hydrated Flux Synthesis. *J. Solid State Chem.* **2014**, *210*, 60–64, doi:10.1016/j.jssc.2013.10.043.
7. Huang, Y.-X.; Prots, Y.; Kniep, R. $Zn[BPO_4(OH)_2]$: A Zinc Borophosphate with the Rare Moganite-Type Topology. *Chem. - Eur. J.* **2008**, *14*, 1757–1761, doi:10.1002/chem.200701349.
8. Zheng, J.; Zhang, A. An Open-Framework Borophosphate, $LiCu_2BP_2O_8(OH)_2$. *Acta Crystallogr., Sect. E: Struct. Rep. Online* **2009**, *65*, i40–i40, doi:10.1107/S1600536809015554.
9. Yang, M.; Yu, J.; Di, J.; Li, J.; Chen, P.; Fang, Q.; Chen, Y.; Xu, R. Syntheses, Structures, Ionic Conductivities, and Magnetic Properties of Three New Transition-Metal Borophosphates $Na_5(H_3O\{M^{II}_3[B_3O_3(OH)]_3(PO_4)_6\}\cdot 2H_2O$ (M^{II} = Mn, Co, Ni). *Inorg. Chem.* **2006**, *45*, 3588–3593, doi:10.1021/ic051916f.
10. Boy, I.; Cordier, G.; Eisenmann, B.; Kniep, R. Oligomere Tetraeder-Anionen in Borophosphaten: Darstellung Und Kristallstrukturen von $NaFe[BP_2O_7(OH)_3]$ Und $K_2Fe_2[B_2P_4O_{16}(OH)_2$. *Z. Naturforsch., B: J. Chem. Sci.* **1998**, *53*, 165–170, doi:10.1515/znb-1998-0207.
11. Menezes, P.W.; Hoffmann, S.; Prots, Y.; Kniep, R. Synthesis and Crystal Structure of $CaCo(H_2O)[BP_2O_8(OH)]\cdot H_2O$. *Z. Anorg. Allg. Chem.* **2009**, *635*, 614–617, doi:10.1002/zaac.200801376.
12. Menezes, P.W.; Hoffmann, S.; Prots, Y.; Kniep, R. Crystal Structure of Calcium Iron(II) Hydrogenmonophosphatedihydrogenmonoborate- Monophosphate, $CaFe[BP_2O_7(OH)_3]$. *Z. Kristallogr. - New Cryst. Struct.* **2008**, *223*, 335–336, doi:10.1524/ncrs.2008.0145.
13. Miao, Z.; Yang, Y.; Wei, Z.; Yang, Z.; Yu, S.; Pan, S. $NaCa_5BO_3(SiO_4)_2$ with Interesting Isolated $[BO_3]$ and $[SiO_4]$ Units in Alkali- and Alkaline-Earth-Metal Borosilicates. *Inorg. Chem.* **2019**, doi:10.1021/acs.inorgchem.9b00002.
14. Barrer, R.M.; Freund, E.F. Hydrothermal Chemistry of Silicates. Part XVIII. Reactions in the System CaO–B_2O_3–SiO_2–H_2O. *J. Chem. Soc., Dalton Trans.* **1974**, 2060–2065, doi:10.1039/DT9740002060.
15. Saradhi, M.P.; Boudin, S.; Varadaraju, U. V.; Raveau, B. A New $BaB_2Si_2O_8$:Eu^{2+}/Eu^{3+}, Tb^{3+} Phosphor – Synthesis and Photoluminescence Properties. *J. Solid State Chem.* **2010**, *183*, 2496–2500, doi:10.1016/J.JSSC.2010.07.008.
16. Netzsch, P.; Pielnhofer, F.; Glaum, R.; Höppe, H.A. Synthesis-Controlled Polymorphism and Optical Properties of Phyllosilicate-Analogous Borosulfates $M[B_2(SO_4)_4]$ (M =Mg, Co). *Chem. - Eur. J.* **2020**, *26*, 14745–14753, doi:10.1002/chem.202003214.
17. Hämmer, M.; Bayarjargal, L.; Höppe, H.A. The First Bismuth Borosulfates Comprising Oxonium and a Tectosilicate-Analogous Anion. *Angew. Chem. Int. Ed.* **2021**, *60*, 1503–1506, doi:10.1002/anie.202011786.
18. Netzsch, P.; Gross, P.; Takahashi, H.; Höppe, H.A. Synthesis and Characterization of the First Borosulfates of Magnesium, Manganese, Cobalt, Nickel, and Zinc. *Inorg. Chem.* **2018**, *57*, 8530–8539, doi:10.1021/acs.inorgchem.8b01234.
